# Targeting the FOXA1/BMI1 axis to overcome chemoresistance and suppress tumor progression in nasopharyngeal carcinoma

**DOI:** 10.1038/s41420-025-02595-6

**Published:** 2025-07-07

**Authors:** Yaping Qin, Mingqing Yang, Yunzhu Cao, Yue Fu, Fan Yang, Xiaoling Zhang, Shengjun Xiao

**Affiliations:** 1 Department of Pathology, The Second Affiliated Hospital of Guilin Medical University, Guilin, China; 2 Department of Clinical Laboratory Services, Liuzhou Workers’ Hospital, Liuzhou, China; 3 Department of Pathology, The Affiliated Hospital of Guilin Medical University, Guilin, China; 4 Department of Physiology, Faculty of Basic Medical Sciences, Guilin Medical University, Guilin, China

**Keywords:** Head and neck cancer, Tumour-suppressor proteins

## Abstract

Nasopharyngeal carcinoma (NPC) is a highly aggressive head and neck cancer characterized by a complex etiology and a propensity for metastasis. The current study explores the intricate relationship between Forkhead Box A1 (FOXA1) and B-cell-specific Moloney murine leukemia virus integration site 1 (BMI1) in the cancer progression and chemoresistance of NPC. Our research identified a significant downregulation of FOXA1 in NPC tissues and cell lines, which correlates with advanced clinical stages and poor differentiation, underscoring its potential role as a tumor suppressor. Functional assays demonstrated that the silencing of FOXA1 significantly enhanced the proliferation, migration, and invasive capabilities of NPC cells in vitro. Furthermore, the deficiency of FOXA1 was associated with a diminished sensitivity to cisplatin, as evidenced by increased cell viability, reduced apoptosis, and impaired cell cycle arrest upon drug exposure. Mechanistic studies revealed BMI1 as a critical downstream target of FOXA1. We observed a negative correlation between the expression levels of FOXA1 and BMI1 in NPC tissues. FOXA1 was shown to bind directly to the BMI1 promoter, effectively dampening its transcriptional activity. Rescue experiments indicated that the downregulation of BMI1 could partially reverse the malignant phenotypes induced by FOXA1 silencing, both in vitro and in vivo. Importantly, the knockdown of BMI1 significantly increased the chemosensitivity of FOXA1-depleted NPC cells to cisplatin, effectively counteracting the drug resistance associated with FOXA1 suppression. These findings highlight the pivotal role of FOXA1 in NPC development and progression and suggest that its loss leads to the upregulation of BMI1 and the acquisition of cisplatin resistance. Our study provides novel insights into the molecular mechanisms underlying the malignancy and chemoresistance of NPC and proposes that targeting the FOXA1/BMI1 axis could offer a promising therapeutic strategy for the treatment of this devastating disease.

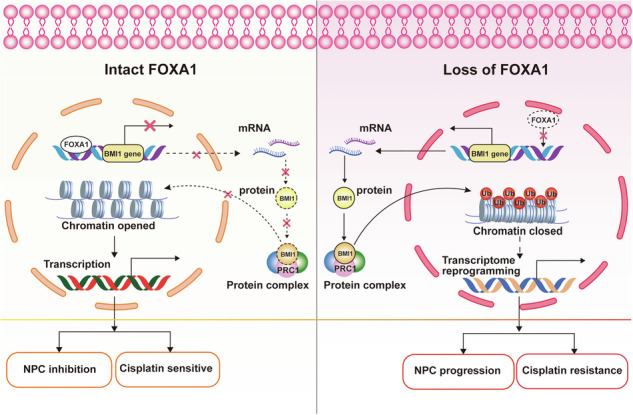

## Introduction

NPC is a unique malignancy of the head and neck, arising from the nasopharyngeal epithelium and exhibiting a significant geographic and ethnic distribution, with a higher incidence noted among populations in Eastern and Southeastern Asia [1]. The disease’s elusive nature within the body and its tendency for early lymphatic spread often result in a majority of patients being diagnosed at an advanced stage [2]. The current standard of care involves concurrent chemoradiotherapy (CCRT), primarily with cisplatin, potentially integrated with induction chemotherapy (IC)or adjuvant chemotherapy (AC) [3, 4]. Advances in intensity-modulated radiotherapy (IMRT) and chemotherapy have improved local control and reduced mortality [5–8]; However, the development of cisplatin resistance poses a formidable barrier to treatment efficacy [9, 10].

The forkhead protein FOXA1, a key winged-helix transcription factor, plays a pioneering role in gene regulation [11], binding to condensed chromatin to enhance accessibility for other transcription factors, thereby influencing a spectrum of genes tied to cell adhesion, cycle, and differentiation—key players in cancer progression [12–15]. Meanwhile, FOXA1, closely linked to tumor chemoresistance, enhances EMT, metastasis, and chemoresistance in docetaxel-resistant LAD cells [16], and its upregulation in endocrine therapy-resistant breast cancer promotes drug resistance via transcriptional regulation of downstream genes [17]. Its downregulation in NPC, as observed in studies, suggests a tumor-suppressive function, with restoration of FOXA1 in NPC cells leading to a suppression of proliferation and invasiveness [18–20]. Yet, the detailed mechanisms of FOXA1 in NPC chemosensitivity remain to be fully elucidated.

BMI1, a linchpin of the Polycomb Group (PcG) proteins, is an essential component of the Polycomb Repressive Complex 1 (PRC1), exerting a pivotal role in epigenetic regulation [21]. BMI1-mediated ubiquitination of histone H2A at lysine 119 within the PRC1 complex is essential for gene silencing, stem cell preservation, and cell cycle regulation [21–23]. BMI1 overexpression in NPC is significantly correlated with heightened malignancy, promoting cancer stem cell self-renewal and tumor invasiveness, thereby adversely impacting patient prognosis [24]. Furthermore, this overexpression is a critical determinant of chemoresistance in NPC, particularly to cisplatin. It diminishes chemosensitivity by inhibiting chemotherapy-induced apoptosis and bolstering cell survival mechanisms [25, 26]. Targeting BMI1 offers a strategic oncological intervention, with its inhibition potentially restoring cisplatin sensitivity in NPC cells, thereby enhancing the efficacy of chemotherapy. This targeted strategy not only addresses the chemoresistance challenge in NPC but also heralds a promising avenue for improving patient outcomes, underscoring BMI1 as an innovative therapeutic target in the fight against drug resistance in cancer treatment.

In this study, we delve into the FOXA1/BMI1 axis within NPC, identifying a pivotal link between diminished FOXA1 expression and BMI1’s role in promoting malignancy and cisplatin resistance. By targeting this axis, we propose a therapeutic strategy to enhance NPC cells’ chemosensitivity to cisplatin and overcome chemoresistance, a major challenge in oncology. Our findings underscore the FOXA1/BMI1 axis as a key target for NPC treatment, offering insights into a potential new approach to improve patient outcomes in this aggressive cancer.

## Results

### FOXA1 is frequently downregulated in NPC tissues and cell lines and its downregulation is associated with aggressive clinicopathological characteristics in NPC

**Fig. 1:**
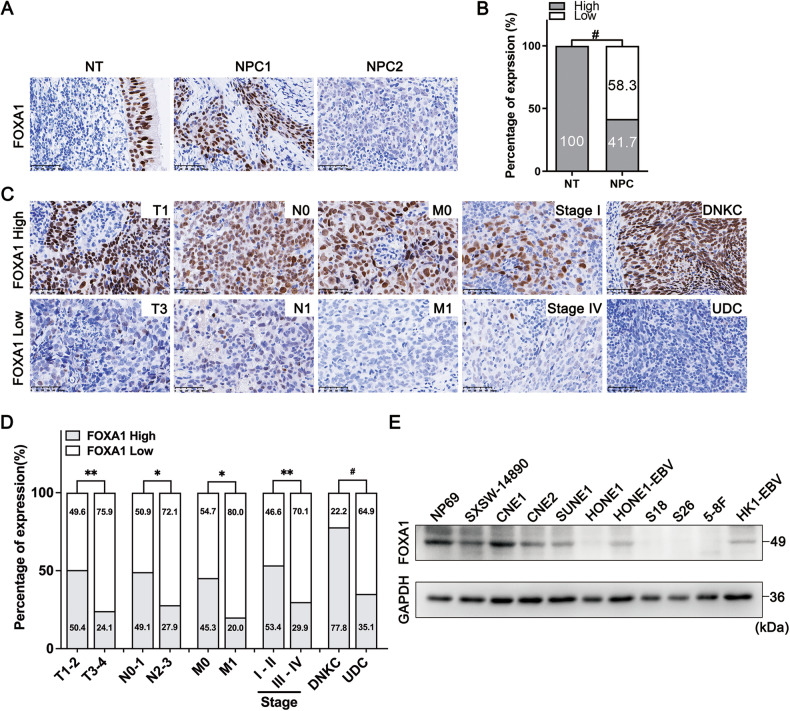
Decreased FOXA1 expression in NPC tissues correlates with advanced clinicopathological features. **A** Representative immunohistochemical staining for FOXA1 protein expression in normal nasopharyngeal epithelium (*n* = 61) and NPC tissues *(n* = 175). Left panel: Normal nasopharyngeal epithelium exhibiting high FOXA1 expression (*n* = 61). Central panel: NPC tissue with high FOXA1 expression (*n* = 73). Right panel: NPC tissue with low FOXA1 expression (*n* = 102). Scale bar = 50 μm. **B** FOXA1 levels were significantly reduced in NPC tissues compared to non-cancerous nasopharyngeal epithelial tissues (*P* < 0.001, Chi-square test). **C** Representative immunohistochemical images of FOXA1 expression in NPC tissues exhibiting a range of clinicopathological features. High FOXA1 expression is observed in tissues from early-stage disease, specifically T1, N0, M0, and stage I, as well as in the differentiated non-keratinizing carcinoma (DNKC) subtype. In contrast, low FOXA1 expression is detected in tissues from advanced stages, characterized by T4, N2, M1, and stage IV, and in the undifferentiated carcinoma (UDC) subtype. Scale bar = 50 μm. **D** Bar graph depicting the stratified analysis of FOXA1 expression levels in NPC tissues, categorized by TNM stage and histological subtype. Statistical differences were evaluated using Chi-square test. **E** FOXA1 protein expression was quantified in designated immortalized nasopharyngeal epithelial cells and NPC cell lines using western blot analysis.

To investigate the influence of FOXA1 in the pathogenesis and tumorigenesis of NPC, we utilized immunohistochemistry to assess FOXA1 protein expression in 175 samples of NPC and 61 samples of non-cancerous nasopharyngeal epithelium (NPE). Uniformly strong nuclear FOXA1 staining was observed in all NPE tissues. Conversely, a significant reduction in FOXA1 protein levels was noted in 58.3% (102/175) of NPC samples (Fig. 1A, B; Table 1). Western blot analysis revealed a significant decrease in FOXA1 expression in most NPC cell lines, including CNE2, SUNE1, HONE1, HONE1-EBV, S18, S26, 5-8 F, and HK1-EBV, compared to the immortalized nasopharyngeal epithelial cell lines NP69 and SWSX-14890 (Fig. 1E), except for CNE1. The consistent downregulation of FOXA1 in NPC tissues, coupled with its notable deficiency in various NPC cell lines, implicates the diminished expression of FOXA1 as a potentially pivotal molecular alteration in the oncogenesis of NPC. These findings suggest FOXA1 downregulation may be a key molecular change in NPC oncogenesis.

**Table 1:** Expression of FOXA1 in 61 non-cancerous epithelial tissues and 175 NPC.

Variables	*n*	FOXA1 expression		χ2	*P*
		Low (*n*, %)	High (*n*, %)		
non-cancerous epithelial tissues	61	0 (0.0)	61 (100.0)	36.842	<0.001
NPC	175	102 (58.3)	73 (41.7)		

Our subsequent analysis showed that FOXA1 expression was significantly inversely correlated with WHO classification and clinical TNM staging. Lower FOXA1 expression was primarily linked to UDC and advanced stages, marked by higher T stage (tumor sizes, T3-T4), N stage (lymph node metastasis, N2-N3), M stage (distant metastasis, M1) and clinical stage (III-IV) (Fig. 1C, D; Table 2). The data above indicate that reduced FOXA1 expression is significantly associated with aggressive NPC traits, suggesting a role in tumor progression.

**Table 2:** Correlation between the clinicopathological features and expression of FOXA1.

Characteristics	Case No. (*n*)	FOXA1 expression		*χ2*	*P*
		High (*n*, %)	Low (*n*, %)		
Sex		73	102		
Female	45	15 (33.3)	30 (66.7)	0.078	0.781
Male	130	58 (44.6)	72 (55.4)		
Age (years)					
<50	81	31 (38.3)	50 (61.7)	0.735	0.443
≥50	94	42 (44.7)	52 (55.3)		
T classification					
T1–T2	117	59 (50.4)	58 (49.6)	11.023	0.001
T3–T4	58	14 (24.1)	44 (75.9)		
N classification					
N0–N1	114	56 (49.1)	58 (50.9)	7.383	0.01
N2–N3	61	17 (27.9)	44 (72.1)		
M classification					
M0	150	68 (45.3)	82 (54.7)	5.656	0.027
M1	25	5 (20.0)	20 (80.0)		
Clinical stage					
I–II	58	31 (53.4)	27 (46.6)	8.831	0.004
III–IV	117	32 (29.9)	75 (70.1)		
Histological type					
DNKC	27	21 (77.8)	6 (22.2)	17.078	<0.001
UDC	148	52 (35.1)	96 (64.9)		

*T* tumor size, *N* lymph node metastasis, *M* distant metastasis, *DNKC* differentiated nonkeratinizing carcinoma, *UDC* undifferentiated carcinoma.

### FOXA1 silencing enhances proliferation, migration and invasion of NPC cells

**Fig. 2:**
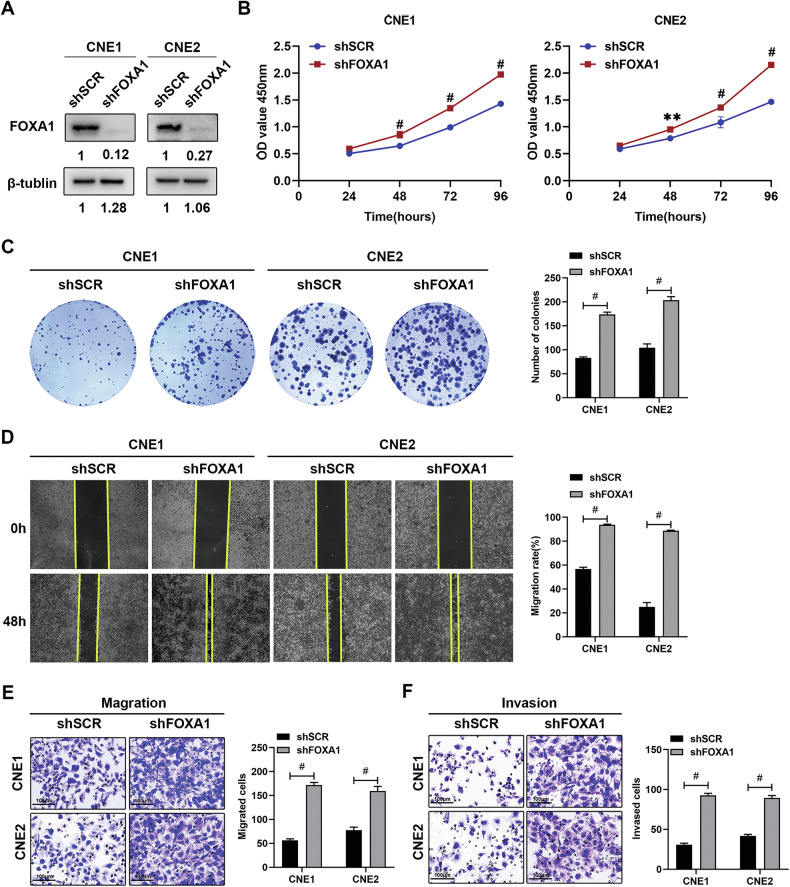
shRNA-mediated FOXA1 knockdown promotes proliferation, migration, and invasion in NPC cells. **A** Efficient knockdown of FOXA1 in CNE1 and CNE2 cells was achieved via lentiviral shRNA delivery, and the corresponding reduction in protein levels was confirmed by Western blot analysis. **B** The CCK-8 assay was employed to assess the impact of FOXA1 knockdown on the growth of CNE1 and CNE2 cells. **C** A colony formation assay was conducted to evaluate the proliferative potential of FOXA1-silenced CNE1 and CNE2 cells. **D** Wound healing assays were utilized to examine the migratory capacity of CNE1 and CNE2 cells following FOXA1 knockdown. **E**, **F** The migratory and invasive capabilities of FOXA1-depleted CNE1 and CNE2 cells were determined using Transwell migration and Matrigel invasion assays, respectively. Data are presented as the mean ± SD from three replicates. **P < 0.01, ^#^*P* < 0.001, as determined by one-way ANOVA test.

To investigate the role of FOXA1 in NPC cells, we utilized lentiviruses encoding FOXA1 shRNA and overexpression constructs to modulate FOXA1 levels in CNE1 and CNE2 cell lines. Western blot analysis confirmed the successful knockdown and overexpression of FOXA1 (Fig. 2A; Supplementary Fig. 2A). Reduction in FOXA1 expression was associated with increased cell proliferation as evidenced by CCK-8 (Fig. 2B) and colony formation assays (Fig. 2C). Additionally, FOXA1 depletion significantly enhanced the migration and invasion of CNE1 and CNE2 cells as shown by wound healing (Fig. 2D) and Transwell assays (Fig. 2E, F). Conversely, overexpression of FOXA1 in CNE1 cells resulted in decreased proliferation, migration, and invasion capabilities (Supplementary Fig. 2B–E). These findings collectively suggest that FOXA1 acts as a negative regulator of cell proliferation, migration, and invasion in NPC cell lines, highlighting its potential as a tumor suppressor and a promising target for NPC therapeutic intervention.

### FOXA1 silencing diminishes cisplatin chemosensitivity in NPC Cells

**Fig. 3:**
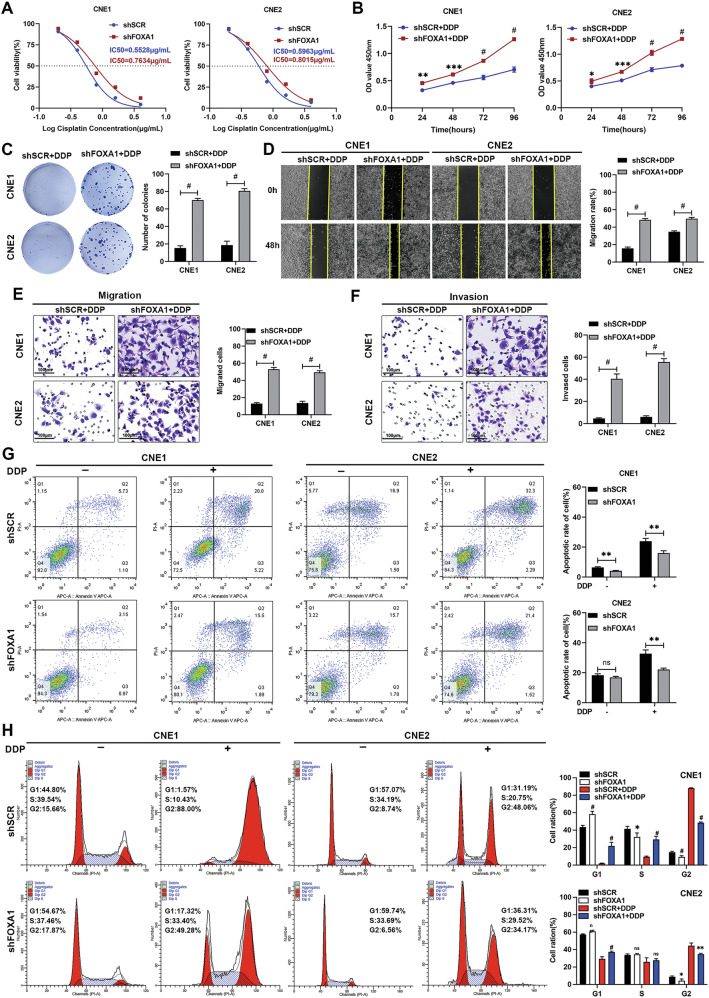
Knockdown of FOXA1 decreased the chemosensitivity of NPC cells to cisplatin in vitro. **A** FOXA1-silenced CNE1 and CNE2 cells were subjected to a series of cisplatin concentrations to ascertain the IC50 values via the CCK-8 assay. Following treatment with cisplatin at 0.5 μg/mL for 48 h, these cells were assessed using: **B** the CCK-8 assay, **C** wound healing assays, (**E**, **F**) Transwell migration and invasion assays, and (**G**, **H**) flow cytometry. Data are presented as the mean ± SD from three replicates. NS, not significant; *P < 0.05, **P < 0.01 and ^#^*P* < 0.001, as determined by one-way ANOVA test.

To elucidate the impact of FOXA1 suppression on NPC cells’ response to cisplatin, we exposed CNE1 and CNE2 cells to varying cisplatin concentrations for 48 h and determined the IC50 values. NPC cells with diminished FOXA1 expression exhibited an elevated IC50, suggesting a lower sensitivity to cisplatin (Fig. 3A). Further analysis using CCK-8 and colony formation assays showed that downregulation of FOXA1 led to a significant rise in cisplatin resistance, evidenced by enhanced cell viability and colony counts (Fig. 3B, C). Additionally, the wound healing and Transwell assays indicated that FOXA1 depletion reduced the inhibitory impact of cisplatin on cell migration and invasion, pointing to a less effective drug response (Fig. 3D–F). Flow cytometry data confirmed that cisplatin’s induction of G2/M cell cycle arrest in CNE1 and CNE2 cells was counteracted by FOXA1 silencing (Fig. 3G). The Annexin V/PI assay also demonstrated that cells with FOXA1 knockdown had a significantly decreased rate of apoptosis in response to cisplatin treatment compared to the control cells (Fig. 3H). These results collectively indicate that diminished FOXA1 expression is associated with a reduced chemotherapeutic response to cisplatin in NPC cells, highlighting the significance of FOXA1 in the modulation of drug sensitivity.

### FOXA1 transcriptionally inhibits the expression of BMI1 in NPC cells

**Fig. 4:**
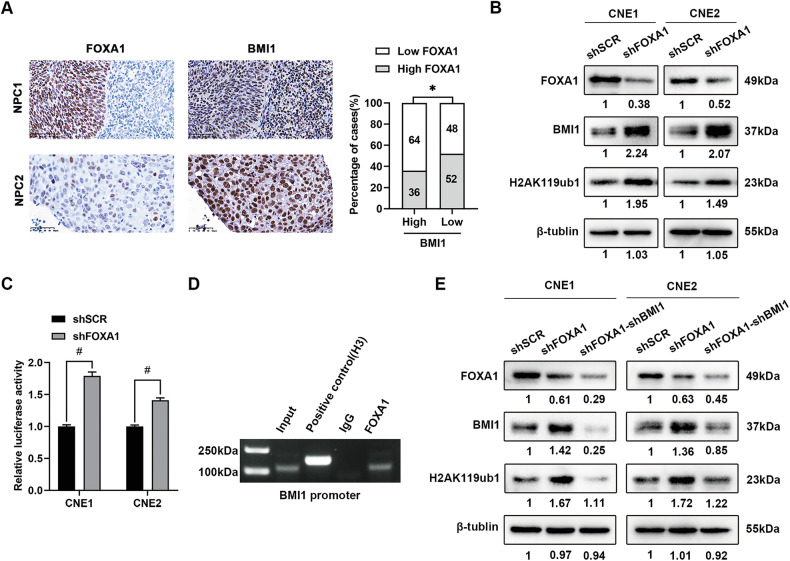
FOXA1 negatively regulates BMI1 expression in NPC. **A** Immunohistochemical (IHC) experiments revealed a significant negative correlation between FOXA1 and BMI1 protein expression in NPC patient samples. Representative images illustrate varying levels of BMI1 expression in NPC tissues corresponding to high or low FOXA1 expression. Scale bar = 50 μm. **B** Following FOXA1 knockdown in CNE1 and CNE2 cells, the protein expression levels of BMI1 and the histone modification marker H2AK119ub1 were assessed using western blot analysis. **C** The impact of FOXA1 knockdown on BMI1 promoter activity was evaluated using a dual-luciferase reporter assay. **D** Chromatin immunoprecipitation polymerase chain reaction (ChIP-PCR) assays confirmed the binding of FOXA1 to a specific region within the BMI1 gene promoter. **E** Western blot analysis revealed an inverse correlation between FOXA1 and BMI1 expression in NPC cells, where FOXA1 knockdown led to increased BMI1 levels and a corresponding elevation of the BMI1-dependent histone modification H2AK119ub1. To elucidate the role of BMI1 in FOXA1-mediated functions, BMI1 knockdown was induced in FOXA1-silenced NPC cells, resulting in a significant reduction of H2AK119ub1, as confirmed by western blotting. Data are represented as the mean ± SD. *P < 0.05 and ^#^*P* < 0.001.

To investigate FOXA1’s role in NPC progression and chemosensitivity, we utilized the hTFtarget database to identify FOXA1-target genes, focusing on BMI1, known for its association with cisplatin resistance in NPC [26]. Bioinformatics analysis implicated BMI1 as a potential transcriptional target of FOXA1 (https://guolab.wchscu.cn/hTFtarget//#!/targets/chipseq_tf?tf=FOXA1). To further delineate the transcriptional regulation of BMI1 by FOXA1, an evaluation of the expression correlation between FOXA1 and BMI1 across NPC tissues and cell lines was conducted. Thereafter, the regulatory influence of FOXA1 on BMI1 transcription was empirically validated through ChIP-PCR and a dual-luciferase reporter gene assay. Immunohistochemical analysis of NPC tissues revealed a significant inverse correlation between FOXA1 and BMI1 expression levels (Fig. 4A). Western blot analysis indicated that upon FOXA1 knockdown, BMI1 protein levels were upregulated in CNE1 and CNE2 cells (Fig. 4B), whereas overexpression of FOXA1 in CNE1 cells resulted in decreased BMI1 levels (Supplementary Fig. 2A). Furthermore, the epigenetic modification, histone H2AK119 monoubiquitination (H2AK119ub1), mediated by the BMI1-associated PRC1 complex, was observed to correlate with the expression levels of BMI1 in FOXA1-knockdown CNE1 and CNE2 cells (Fig. 4B). Conversely, H2AK119ub1 was downregulated in FOXA1-overexpressing CNE1 cells (Supplementary Fig. 2A). These findings suggest that BMI1-mediated epigenetic effects are associated with BMI1 expression levels in NPC cells, although further studies are needed to elucidate the underlying mechanisms. To reinforce the transcriptional control of BMI1 by FOXA1, a luciferase reporter assay incorporating a FOXA1-responsive element in the BMI1 promoter was conducted. Transfection of CNE1 and CNE2 cells with this reporter alongside LV-FOXA1 or LVcon, followed by incubation, resulted in diminished luciferase activity with LV-FOXA1, thereby validating FOXA1’s enhancer function on the BMI1 promoter (Fig. 4C). ChIP assays confirmed specific FOXA1 binding to the BMI1 promoter sequence, in contrast to the non-specific IgG control (Fig. 4D). These findings collectively demonstrate FOXA1’s direct interaction with and regulation of the BMI1 promoter in NPC cells.

### BMI1 downregulation abrogates the malignant phenotypes conferred by FOXA1 suppression in NPC cells

**Fig. 5:**
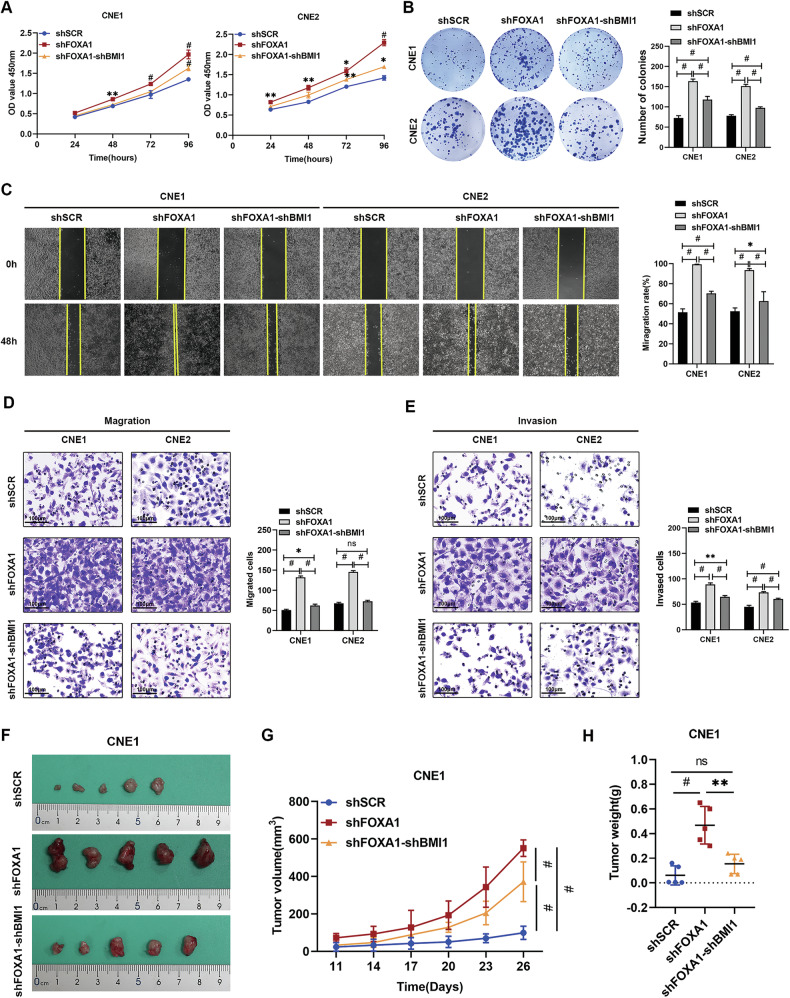
BMI1 knockdown abrogates the tumor-promoting effects conferred by FOXA1 knockdown in NPC cells. CNE1 and CNE2 cells were transfected with lentiviral shRNA constructs targeting scrambled (shSCR), FOXA1 (shFOXA1), or dual-targeting FOXA1 and BMI1 (shFOXA1-shBMI1) sequences. Subsequent analyses included: **A** CCK-8 assay for cell viability, **B** Colony formation assay for proliferative capacity, **C** Wound healing assay for migration, and **D**, **E** Transwell assays for migration and invasion, respectively. In the xenograft model, CNE1 cells were injected into nude mice, with tumor volumes monitored every three days. Results are depicted in **F** a tumor photograph, **G** tumor growth curve, and **H** tumor weight measurements. Data are presented as Mean ± SD with three replicates. Statistical significance was determined using one-way ANOVA followed by Tukey’s multiple comparisons test. NS not significant; *P < 0.05, **P < 0.01 and ^#^*P* < 0.001.

To clarify the molecular interplay between FOXA1 and BMI1 in NPC, rescue assays were conducted by specifically reducing BMI1 levels in CNE1 and CNE2 cells after the initial knockdown of FOXA1. Western blot analysis confirmed the successful knockdown of BMI1 in FOXA1-silenced NPC cells, accompanied by a reduction in the level of its epigenetic effector, H2AK119ub1, which had been upregulated upon FOXA1 silencing (Fig. 4E). The CCK-8 assay (Fig. 5A) and colony formation assay (Fig. 5B) demonstrated that the enhanced cell viability observed in the FOXA1 knockdown group was significantly reversed by the additional knockdown of BMI1. Meanwhile, the wound healing assay (Fig. 5C) and Transwell assay (Fig. 5D, E) showed that BMI1 knockdown effectively mitigated the enhanced migratory and invasive capacities induced by FOXA1 knockdown in CNE1 and CNE2 cells. Moreover, the xenograft tumor model (Fig. 5F–H) showed that the increased tumor growth rate, consequent to FOXA1 knockdown, was significantly reversed by the knockdown of BMI1. These findings highlight the critical role of the FOXA1-BMI1 regulatory axis in the progression of NPC, indicating that the inhibition of BMI1 could offer a promising therapeutic approach for treating NPC, particularly in cases with diminished FOXA1 activity.

### BMI1 downregulation reverses FOXA1-silencing-induced cisplatin resistance in NPC cells

**Fig. 6:**
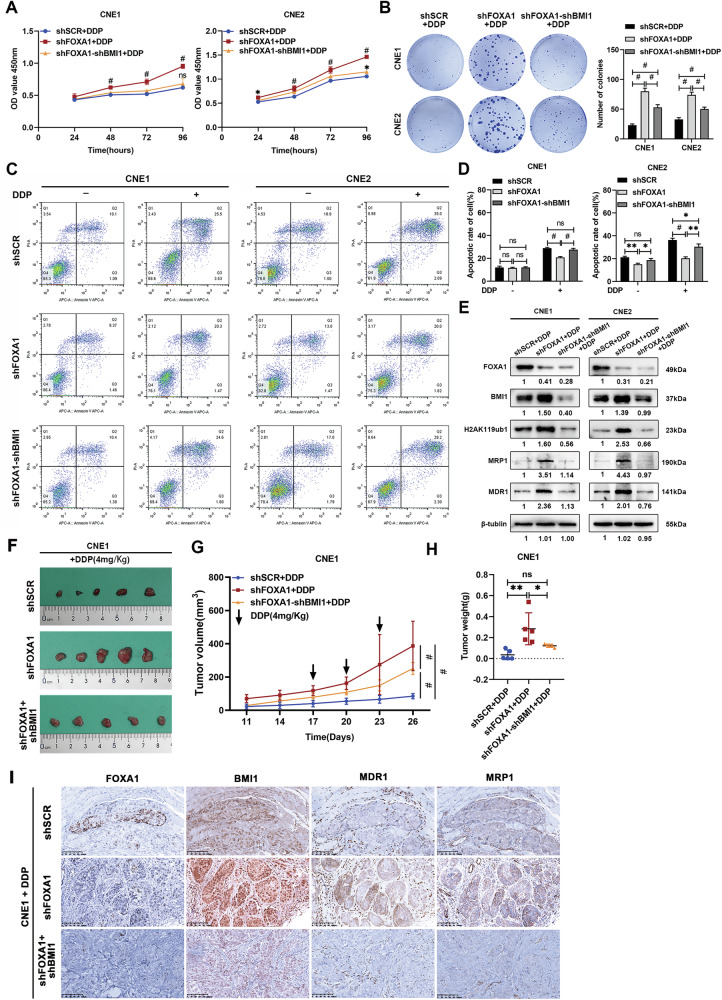
BMI1 downregulation reverses FOXA1-silencing-induced cisplatin resistance in NPC cells. CNE1 and CNE2 cell lines were transduced with plasmids containing shRNA constructs (shSCR for scrambled control, shFOXA1 for FOXA1 knockdown, and shFOXA1+shBMI1 for combined FOXA1 and BMI1 knockdown). Post-transduction, cells were exposed to cisplatin at a concentration of 0.5 μg/mL for subsequent assays. The effects of cisplatin on cell viability were assessed using the CCK8 assay (**A**), and the colony-formation ability was evaluated through the colony-formation assay (**B**). Apoptosis induction was measured by flow cytometry (**C**, **D**). Expression levels of proteins associated with drug resistance were determined by Western blotting (**E**). Nude mice bearing xenografts of the aforementioned cell lines were administered cisplatin at a dosage of 4 mg/kg, with treatments administered every three days for a total of three doses. Tumor growth was monitored, and representative images of the tumors (**F**), tumor growth curves (**G**), and tumor weights (**H**) are presented. Expression of drug resistance-related proteins in the xenograft tissues was further analyzed by immunohistochemistry (**I**). Scale bar = 100 μm. Data are presented as Mean ± SD with three replicates. Statistical significance was determined using one-way ANOVA followed by Tukey’s multiple comparisons test. NS, not significant; *P < 0.05, **P < 0.01 and ^#^*P* < 0.001.

To elucidate the role of BMI1 in FOXA1-mediated cisplatin sensitivity in NPC, we performed in vitro assays to evaluate cell viability (Fig. 6A, B), migration (Supplementary Fig. 1A, B), and invasiveness (Supplementary Fig. 1C) after BMI1 knockdown in cells with silenced FOXA1. These assays revealed that BMI1 knockdown significantly increased cisplatin sensitivity, as evidenced by reduced cell viability, migration, and invasiveness, suggesting an amelioration of chemoresistance associated with FOXA1 deficiency. Concurrently, FOXA1 knockdown was found to decrease cisplatin-induced apoptosis (Fig. 6C) and G2/M cell cycle arrest (Supplementary Fig. 1D), effects that were markedly reversed by BMI1 knockdown. In direct contrast, overexpression of FOXA1 in CNE1 cells, as measured by the aforementioned assays, inhibited malignant phenotypes and enhanced sensitivity to cisplatin, an effect that was counteracted by the concurrent overexpression of BMI1 (Supplementary Figs. 3 and 4). Moreover, Western blotting validated that BMI1 knockdown mitigated the chemoresistance-associated upregulation of MDR1 and MRP1 proteins induced by FOXA1 knockdown (Fig. 6E). Conversely, in CNE1 cells overexpressing FOXA1, the levels of MDR1 and MRP1 proteins were observed to be diminished with BMI1 overexpression (Supplementary Fig. 4C). In the xenograft model, the co-knockdown of FOXA1 and BMI1 in CNE1 cells resulted in an increased sensitivity to cisplatin, as indicated by reduced tumor size (Fig. 6F, H) and decelerated growth rates (Fig. 6G), in stark comparison to the FOXA1-knockdown cells that displayed resistance. In xenografted CNE1 cells with combined FOXA1 and BMI1 knockdown, immunohistochemical analysis revealed a reduction in the overexpression of MDR1 and MRP1 proteins, which are associated with FOXA1 knockdown alone (Fig. 6I). The collective findings emphasize the significance of the FOXA1-BMI1 interaction in regulating cisplatin sensitivity in NPC, suggesting that targeting this axis could be key to enhancing chemosensitivity and optimizing treatment efficacy.

## Discussion

NPC is an aggressive head and neck cancer, and radiotherapy is a well-established effective treatment for its early stages [1, 27, 28]. The high invasiveness of NPC, which often leads to lymph node infiltration and distant metastasis, and is frequently diagnosed at an advanced stage, results in a poor prognosis and a higher recurrence rate [29, 30]. Advanced NPC is primarily treated with cisplatin-based chemotherapy [3, 4]. However, the development of resistance to cisplatin poses a significant obstacle to successful therapy [10, 31]. The propensity for chemoresistance underscores the urgent need for novel therapeutic strategies that can overcome resistance mechanisms and improve patient outcomes.

The transcription factor FOXA1 has been extensively studied for its role in tumorigenesis and cancer progression, exhibiting context-dependent functions that can range from promoting to suppressing tumor growth [30, 32, 33]. In various cancer types, FOXA1 has been implicated as an oncogene. Its overexpression is significantly associated with the oncogenesis, development, and prognosis of several cancers, including breast, prostate, and lung [33–36]. In the context of NPC, a highly malignant cancer often diagnosed at advanced stages, FOXA1 appears to play a protective role. Prior studies have reported significant downregulation of FOXA1 in NPC tissues and cell lines, correlating with advanced clinical stages and poor differentiation, which suggests its potential as a tumor suppressor [18–20]. Utilizing a larger cohort of 175 NPC and 61 non-cancerous nasopharyngeal epithelia cases, we demonstrated that loss of FOXA1 was significantly associated with larger tumor size, lymphatic metastasis, distant metastasis, advanced clinical stages, and the undifferentiated histological subtype. In NPC cells and corresponding xenografts, assays indicated that FOXA1 functions as a tumor suppressor, with its knockdown promoting malignant behaviors such as increased cell proliferation, migration, and invasion. This research not only supports the notion of FOXA1 as a tumor suppressor, in line with previous studies, but also underscores its critical role in tumor progression.

Furthermore, FOXA1 is reported to play an essential role in chemoresistance, with high levels contributing to chemoresistance in lung and cervical cancers [16, 37]. However, in estrogen receptor-positive breast cancer, FOXA1 downregulation enhances sensitivity to doxorubicin and paclitaxel, while its upregulation in basal-like breast cancer cells confers increased drug resistance. These findings establish FOXA1 as a context-dependent modulator of chemoresistance, with its regulatory effects contingent upon specific cellular microenvironments. Moreover, our study demonstrated that FOXA1 silencing in NPC cells enhanced cisplatin resistance, as evidenced by increased proliferative capacity, migratory/invasive potential, and reduced cisplatin-induced apoptosis and cell cycle arrest. This finding contrasts with a previous report documenting FOXA1 upregulation in the cisplatin-resistant NPC cell line CNE2/DDP, where FOXA1 knockdown conversely improved cisplatin sensitivity [38]. This discrepancy may stem from inherent limitations in the CNE2/DDP model system, where prolonged drug exposure has induced extensive genetic alterations that could confound mechanistic interpretations. While these conflicting results highlight the potential context-dependent roles of FOXA1 in chemoresistance, the biological validity of FOXA1’s opposing effects observed in chronically drug-adapted cell lines requires further investigation through multi-model validation studies.

FOXA1, utilizing its intrinsically disordered regions (IDRs) to mediate phase separation via the formation of biomolecular condensates, enables the binding to and unpacking of condensed chromatin [39]. By facilitating the opening of these specific chromatin regions, FOXA1 acts as a “pioneer” transcription factor, collaborating with a variety of transcription factors and co-factors to regulate the expression profile of target genes. In prostate cancer, FOXA1’s interaction with the androgen receptor (AR) enhances AR-mediated transcription, potentially driving tumor progression and castration resistance [35]. In breast cancer, the upregulation of FOXA1 leads to the reprogramming of estrogen receptor function, contributing to endocrine resistance and the promotion of metastasis in ER-positive tumors through a High-FOXA1/ER-dependent secretory mechanism [40]. FOXA1 directly binds to the MND1 promoter to suppress its transcription, and this suppression-mediated activation of the PI3K/AKT signaling axis concomitantly inhibited gastric cancer progression and enhanced oxaliplatin chemosensitivity [41]. Collectively, FOXA1 can alter the affinity of specific cis-regulatory elements for their associated transcription factors, thereby regulating the expression of target gene profiles, leading to changes in the malignant biological behaviors and therapeutic responses of tumors.

In NPC, FOXA1 regulates a range of target genes, including the oncomiRs miR-100-5p and miR-125b-5p [20]. It reprograms the TGF-β-stimulated transcriptional program from promoting metastasis to suppressing tumor progression, thereby restoring NPC cells’ sensitivity to the growth-inhibitory effects of TGF-β [19]. FOXA1 also directly binds to the promoters of EMT-related genes like Slug and recruits co-repressors such as HDAC2 to inhibit their expression, thereby suppressing the EMT [42]. Additionally, FOXA1 enhances antitumor immunity and reduces immune evasion in NPC by interacting with STAT1 to inhibit IRF1 transcription, thereby suppressing IFN-γ-induced PD-L1 expression [43]. These findings establish FOXA1 as a multidimensional regulator of NPC pathogenesis and therapeutic resistance, functioning through both autonomous transcriptional control and cooperative chromatin remodeling with transcriptional co-factors. In our study, by mining the FOXA1 downstream target gene set from the JASPAR database, we identified BMI1 as a potential transcriptional target of FOXA1. Concurrently, a significant inverse correlation between the expression levels of FOXA1 and BMI1 was observed in NPC tissues and in NPC cells. Utilizing ChIP-PCR and luciferase reporter assays, we documented that FOXA1 binds to the promoter region of BMI1 and negatively modulates its transcription.

As a core PRC1 component within the PcG family, BMI1 catalyzes H2AK119 ubiquitination to enforce transcriptionally repressive chromatin states through chromatin compaction [21, 44, 45]. This PRC1-mediated epigenetic remodeling underlies BMI1’s dual regulatory capacity in maintaining stem cell pluripotency and driving oncogenic progression, with emerging roles in therapy-resistant malignancies [22]. BMI1 has been functionally characterized as a pleiotropic oncogene across diverse cancer types, driving tumor progression and therapeutic resistance through epigenetic modulation of specific transcriptional programs [21]. In neuroblastoma, PTC596-induced BMI1 inhibition depletes glutathione, enhances peroxide production and lipid peroxidation, and triggers ferroptosis [46]. In leukemia, hypoxia-induced BMI1 activation promotes chemoresistance in leukemia stem cells by activating the PI3K/Akt pathway and inducing EMT [47]. In gastric cancer, BMI1 upregulates miR-27a and miR-155 to target RKIP, thereby promoting metastasis and chemoresistance [48]. In non-small cell lung cancer, the BMI1/MALAT1 axis sequesters miR-145-5p to support tumor survival [49]. In head and neck squamous cell carcinoma, BMI1 sustains cancer stem cell self-renewal and activates AP-1 transcription, driving metastasis and chemoresistance [50]. These findings indicate that BMI1 promotes tumorigenesis and chemoresistance through diverse mechanisms, including redox balance modulation, chromatin remodeling, and miRNA network regulation, positioning it as a promising therapeutic target.

BMI1 is significantly overexpressed in NPC and correlates with advanced tumor stage, metastasis, and poor prognosis [24]. Functionally, silencing BMI1 inhibits NPC cell proliferation, stemness, motility, and invasion, as evidenced by multiple studies [24, 26, 51]. Mechanistically, BMI1 drives tumor aggressiveness by transcriptionally repressing PTEN, thereby activating the PI3K/Akt pathway, which induces EMT and promotes metastatic dissemination [52]. In CD44 + NPC cancer stem cells (CSCs), BMI1 knockdown attenuates self-renewal, proliferation, migration, and invasion, while concurrently enhancing chemosensitivity to cisplatin and 5-fluorouracil through reactivation of the p16INK4a-p14ARF-p53 tumor suppressor axis [26]. Furthermore, targeting BMI1 augments radiosensitivity by exacerbating DNA damage accumulation and apoptosis induction, highlighting its dual role in overcoming both chemoresistance and radioresistance [51]. Collectively, these findings underscore BMI1 as a pivotal therapeutic target in NPC, offering a strategic avenue to disrupt tumor progression, metastasis, and therapy resistance while improving clinical outcomes.

Given BMI1’s central role in NPC malignancy and drug resistance, this study investigates whether BMI1, validated as a transcriptional target of FOXA1 in our research, mediates FOXA1’s effects on NPC progression and cisplatin resistance. Our results showed that knockdown of BMI1 in NPC cells with endogenous FOXA1 silenced reverses the malignant phenotypes and cisplatin resistance linked to FOXA1 suppression. Concomitant alterations in BMI1 expression and H2AK119ub1 levels (a repressive chromatin marker catalyzed by BMI1 as the core enzymatic component of PRC1) were observed following FOXA1 knockdown and overexpression in NPC cells. This suggests that FOXA1 regulates BMI1 expression, thereby influencing BMI1-driven chromatin remodeling. Our findings indicate that FOXA1 not only directly participates in chromatin remodeling but also exerts an indirect effect through its target. The systematic identification and comprehensive characterization of FOXA1 transcriptional targets in NPC is essential to delineate its functional contributions to tumor progression and therapeutic resistance, while uncovering the molecular mechanisms that orchestrate these pathological processes.

Collectively, these results establish the FOXA1/BMI1 axis as a critical regulator in NPC. FOXA1 acts as a tumor suppressor, and its loss promotes NPC progression and cisplatin resistance, partially through BMI1-mediated mechanisms. Intriguingly, FOXA1 and BMI1 exhibit opposing roles in modulating the PI3K/Akt pathway: BMI1 activates PI3K/Akt signaling to drive tumor growth and chemoresistance [52], whereas FOXA1 suppresses MND1 to inhibit progression and enhance oxaliplatin sensitivity via the same pathway [41]. This functional antagonism suggests that FOXA1 and BMI1 may competitively regulate PI3K/Akt activity, potentially through shared downstream effectors (e.g., Akt) or epigenetic modulation of overlapping targets. For instance, BMI1-driven PI3K/Akt activation could attenuate FOXA1’s tumor-suppressive effects, thereby influencing tumor aggressiveness and therapeutic outcomes. However, the molecular basis of FOXA1/BMI1 crosstalk within this pathway remains unclear, particularly whether their interaction involves direct competition for Akt binding or co-regulation of epigenetic modifiers.

In conclusion, our study demonstrates that FOXA1 drives NPC progression and cisplatin resistance via transcriptional regulation of BMI1, expanding its epigenetic role as a modulator of chromatin remodeling. Targeting the FOXA1/BMI1 axis provides a novel strategy for clinically management of NPC progression and chemoresistance. However, the comprehensive profile of FOXA1’s transcriptional targets in NPC is still unclear, and its mechanisms in regulating chromatin remodeling and interactions with other epigenetic factors need further exploration.

## Materials and methods

### Patient specimens

Formalin-fixed, paraffin-embedded non-cancerous nasopharyngeal tissues (61 cases) and NPC tissues (175 cases), along with their corresponding clinical and pathological data, were collected from the Second Affiliated Hospital of Guilin Medical University (Guilin, China) between 2015 and 2022. The study was approved by the Research Ethics Committee and conducted with patient consent (Approval No. EFY-GZR2022007).

### Hematoxylin-Eosin (H&E) staining and immunohistochemistry (IHC)

Hematoxylin-eosin (H&E) staining [53] and immunohistochemical assays [54, 55] were performed as described previously. Immunohistochemical scores were categorized as 0 (negative), 1 (weak), 2 (moderate), or 3 (strong), based on staining intensity. Scores of 0–1 were considered low expression, while 2–3 indicated high expression. The antibodies utilized for IHC are detailed in Supplementary Table 1.

### Cell lines and culture

Human NPC cell lines, including CNE1, CNE2, SUNE-1, HONE1, HONE1-EBV, S18, S26, 5-8 F, and HK1-EBV, as well as immortalized nasopharyngeal epithelial cell lines NP69 and SXSW-1489, were generously provided by Prof. Dong Xiao of the Institute of Cancer Research, Southern Medical University, Guangzhou, China. The cells were routinely cultured in RPMI-1640 medium (Gibco, California, USA) supplemented with 10% fetal bovine serum (Excellbio, Shanghai, China) and 1% penicillin/streptomycin (Biosharp, Beijing, China). Cultures were maintained in a humidified incubator at 37 °C with an atmosphere of 5% CO_2_.

### Stable cell line generation via lentiviral transduction

Lentiviral particles, including shRNA-expressing constructs targeting FOXA1 (sc-37930-V) and BMI1 (sc-29814-V), as well as overexpression constructs for FOXA1 (sc-400743-LAC) and BMI1 (sc-417606-LAC), were procured from Santa Cruz Biotechnology (Shanghai, China). Control lentiviral particles comprised shRNA (sc-108080) and Activation Particles (sc-437282). Transfection was performed using the manufacturer’s recommended protocol, followed by puromycin selection.

### Western blotting

Western blot analysis was performed according to established protocols [10, 56]. Primary antibodies are detailed in Supplementary Table 1. Protein expression was quantified using ImageJ software.

### Cisplatin treatment

To determine the IC50 values of cisplatin in CNE1 and CNE2 cells and select an appropriate concentration for further chemosensitivity assays, NPC cells were treated with varying concentrations of cisplatin (0.0–4.0 μg/mL; QiLu Pharmaceutical, Jinan, China, Cat. No. H20023461) for 48 h, followed by CCK-8 assay as described [10].

### CCK-8 and Plate colony‑forming assays

The CCK-8 assay and the plate colony-forming assay were performed as previously described [10, 56].

### Wound healing

The wound healing assay was conducted as reported previously [53].

### Transwell migration and invasion assays

NPC cells (1 × 10^5^ or 2 × 10^5^) in serum-free medium were seeded into the upper 8-μm-pore transwell chambers (Corning, NY, USA) with or without Matrigel (BD Biosciences, Bedford, MA, USA). The lower chambers received 500 µL medium with 10% FBS. After 48-h incubation, migrated or invaded cells on the membranes were fixed, stained, and observed under a microscope [53, 56].

### Flow cytometry

Flow cytometry procedures were previously detailed [10, 48]. For cell cycle analysis, cells exposed to cisplatin for 48 h were fixed in 75% ethanol at 4 °C overnight. Post-centrifugation, they were stained with 500 μL propidium iodide (PI) staining buffer (Life-iLab Biotech, Shanghai, China) for 30 min at room temperature in darkness. The cell cycle distribution was determined using a flow cytometer (BD Biosciences, San Jose, CA, USA). For the apoptosis assay, cells treated with cisplatin for 48 h were stained with Annexin V-647 and PI for 15 min in the dark prior to analysis on a FACS Calibur cytometer (BD Biosciences, San Jose, CA, USA).

### Chromatin immunoprecipitation (ChIP) and PCR

The SimpleChIP™ Enzymatic Chromatin IP Kit (Cell Signaling Technology, Cat. No. 9003S) was employed for ChIP analysis, following the manufacturer’s protocol. CNE1 cells were cross-linked with 1% formaldehyde and the reaction was quenched with Glycine. DNA fragments were extracted after digestion with micrococcal nuclease and subsequent ultrasonication. The lysate was immunoprecipitated using anti-FOXA1, normal IgG, or anti-Histone H3 antibodies conjugated to magnetic beads (the information of antibodies listed in Supplementary Table 1). Enrichment of the BMI1 promoter in the precipitates was assessed by PCR with the following primers: BMI1-forward, 5’-TCTACAGGAGAGCGTCACAT-3’ and BMI1-reverse, 5’-ACTTAGCCCGAAACCGTCAG-3’.

### Dual luciferase reporter assay

A potential binding site for the BMI1 promoter with FOXA1 binding sequence was obtained from Jaspar (http://jaspar.genereg.net/), and the binding region was PCR amplified and cloned into the pGL3 vector (Promega, Madison, WI, USA) to obtain a Promoter luciferase reporter vector. The above reporter vectors were transfected into LVcon or LV-FOXA1-infected CNE1 and CNE2 cells, using Lipofectamine^TM^ 3000 (Invitrogen, Carlsbad, California, USA) as per the manufacturer’s protocol. After 24 h, cells were lysed and luciferase and Renilla signals were quantified using the Dual Luciferase Reporter Assay Kit (Promega, Madison, WI, USA) following the manufacturer’s protocol.

### Generation of the subcutaneous xenograft model in mice

Male BALB/c nude mice (6–8 weeks old, 18–22 g; Hunan Silaikejingda, Changsha, China) were maintained under specific-pathogen-free (SPF) conditions. Subcutaneous injections of 5 × 10^6^ CNE1 cells expressing shSCR, shFOXA1, or shFOXA1-shBMI1 were administered in the right axillary region. Tumor dimensions were measured every three days from day ten post-inoculation using a vernier caliper, with tumor volume calculated as 1/2 × width^2^ × length. When tumors reached approximately 100 mm³, mice were treated with cisplatin at 4 mg/kg, with intraperitoneal injections given every three days for a total of three treatments. Euthanasia via cervical dislocation was performed three days after the final treatment, after which the primary tumors were excised and weighed. This study was conducted in compliance with the animal welfare guidelines of the Institutional Animal Care and Use Committee (IACUC) of Guilin Medical University (Guilin, China) and was approved under protocol number GLMC-2022011011.

### Statistical analysis

Quantitative data are presented as mean ± standard deviation (SD). The association of clinicopathological features with FOXA1 expression, as well as the correlation between FOXA1 and BMI1 expression levels, was examined using the chi-square (χ^2^) test. Statistical significance was determined using a one-way analysis of variance (ANOVA) test. Significance was denoted as not significant (NS) for *P* > 0.05, * for *P* < 0.05, ** for *P* < 0.01, # for *P* < 0.001.

## Supplementary information

Supplementary Material

Original full length western blots

## Data Availability

The datasets pertaining to the binding of FOXA1 to the promoter region of the BMI1 gene are available in the JASPAR database (http://jaspar.genereg.net/). Additional datasets generated and analyzed during the current study are available from the corresponding author upon reasonable request. The original full-length western blots are included in the supplemental material.
