# Supplementary material for: Targeting the FOXA1/BMI1 axis to overcome chemoresistance and suppress tumor progression in nasopharyngeal carcinoma
Source: Cell Death Discov. 2025 Jul 7;11:311. doi: 10.1038/s41420-025-02595-6 (PMC12234731; doi:10.1038/s41420-025-02595-6)
Supplement: Supplementary file 1 — Supplementary Material [file 41420_2025_2595_MOESM1_ESM.doc]

**Supplementary Material**

**
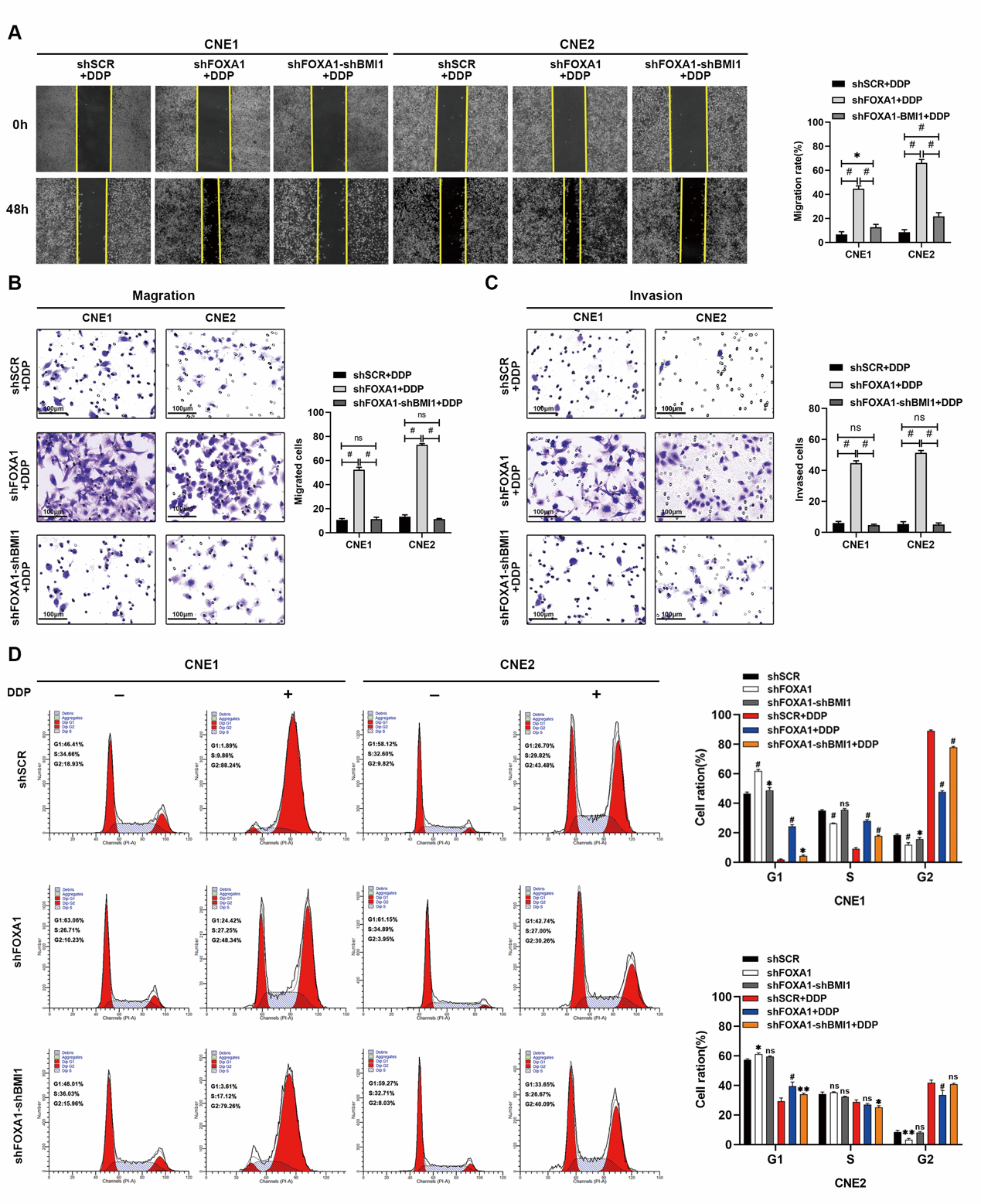
Supplementary Figure 1.** **Silencing BMI1 partially reverses the inhibitory effects of FOXA1 knockdown on cisplatin-induced cell migration, invasion and cell cycle arrest**

Cell migration and invasive capabilities were assessed using the wound healing assay (A) and the Transwell assay (B and C). Cell cycle analysis was performed using flow cytometry (D). Scale bar = 100 μm. Data are presented as the mean ± SD from three independent experiments. Statistical analysis was conducted using one-way ANOVA. NS, not significant; ^*^*P* < 0.05, ^**^*P* < 0.01 and ^#^*P*<0.001.


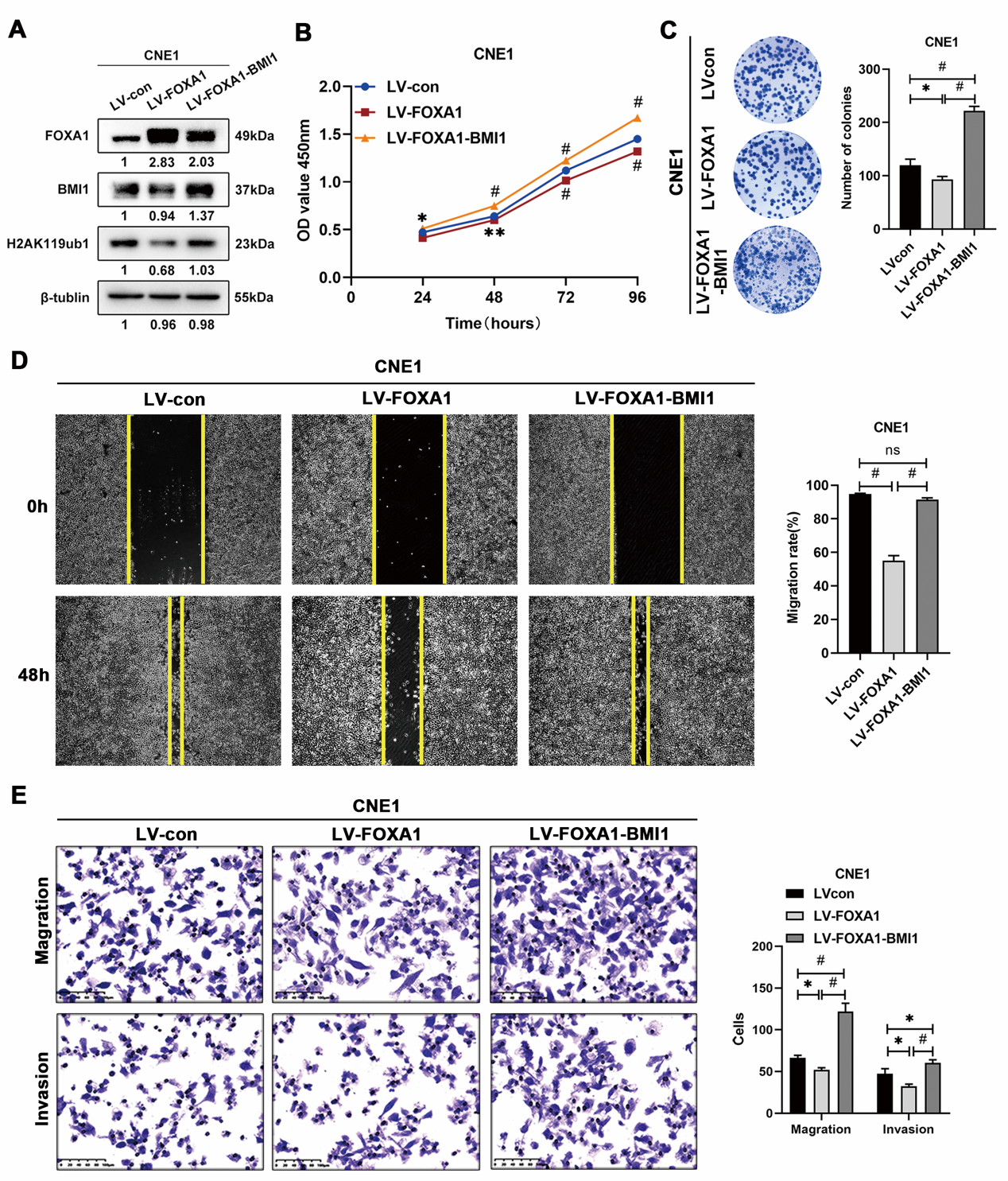


**Supplementary Figure 2. Overexpression of BMI1 partially reverses the inhibitory effects of FOXA1 overexpression on cell proliferation, migration, and invasion.**

In our study of BMI1's influence on FOXA1-induced cellular phenotypes in CNE1 cells, we utilized lentiviral constructs for targeted gene overexpression, including a control with a scrambled sequence (LVcon), FOXA1 alone (LV-FOXA1), and a dual construct for FOXA1 and BMI1 co-overexpression (LV-FOXA1-BMI1). Overexpression at the protein level was verified using Western blot analysis (A). Cellular proliferation was then assessed using the CCK8 assay (B) and the colony formation assay (C). Cell migration and invasion were further evaluated using the wound healing assay (D) and the Transwell assay (E and F), respectively. Scale bar = 100 μm. Data are presented as the mean ± SD from three independent experiments. Statistical analysis was conducted using one-way ANOVA. NS, not significant; ^*^*P* < 0.05, ^**^*P* < 0.01 and ^#^*P*<0.001.


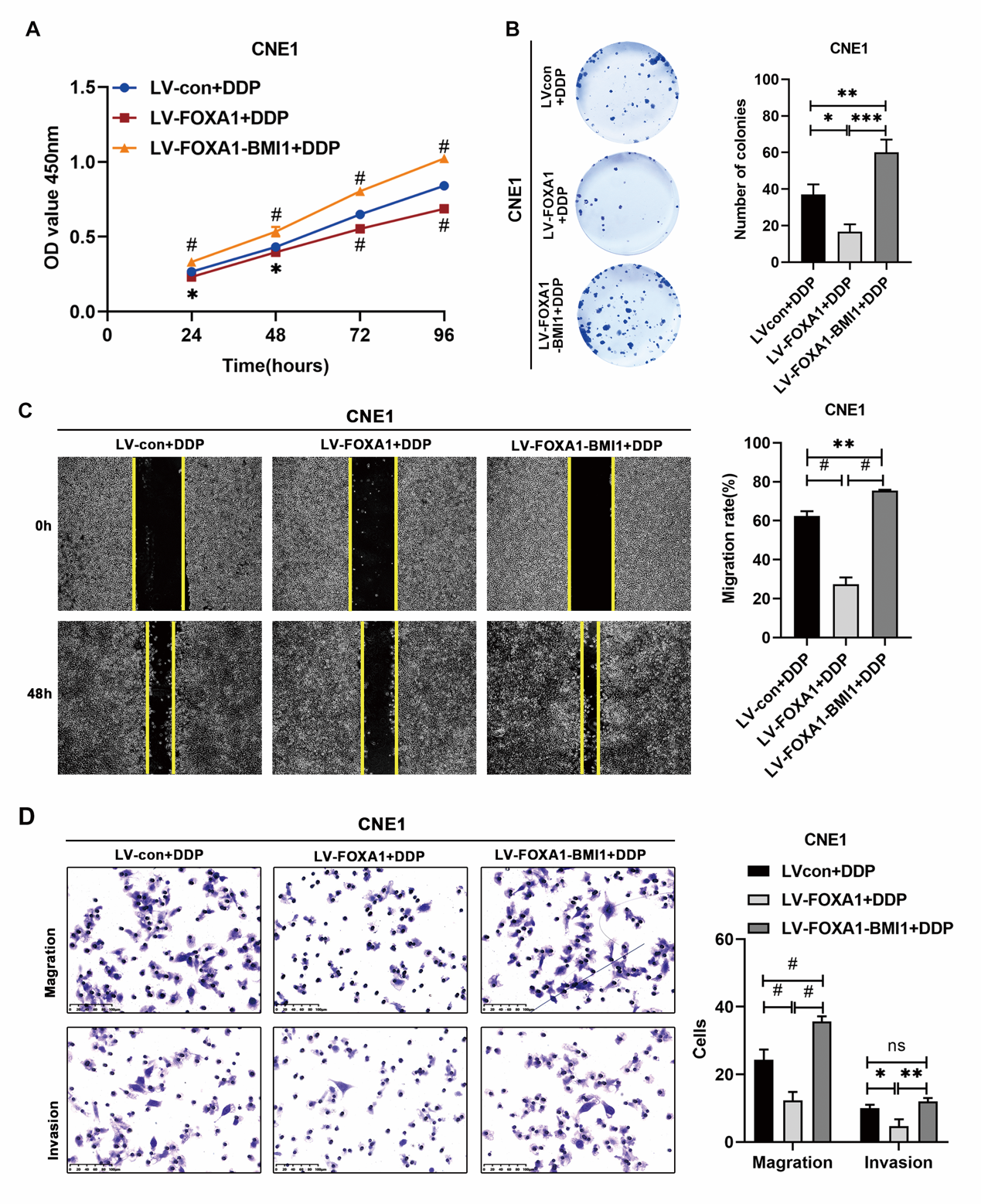


**Supplementary Figure 3. The overexpression of BMI1 partially counteracts the suppressive effect of FOXA1 overexpression, resulting in cisplatin resistance in CNE1 cells.**

CNE1 cells with either overexpression of FOXA1 or co-overexpression with BMI1 were treated with a serial dose of cisplatin Then, these cells were collected for CCK8 assay and subjected to measure the IC50 values of cisplatin (A). Cell viability was measured using the CCK8 assay (B) and the colony formation assay (C). The migration and invasive abilities of the cells were determined using the wound healing assay (D) and the Transwell assay (E, F). Scale bar = 100 μm. Data are presented as the mean ± SD from three independent experiments. Statistical analysis was conducted using one-way ANOVA. NS, not significant; ^*^*P* < 0.05, ^**^*P* < 0.01 and ^#^*P*<0.001.





**Supplementary Figure 4. Overexpression of BMI1 partially reverses the cisplatin-induced cell cycle arrest and apoptosis that is promoted by FOXA1 overexpression, and also reverses the downregulation of genes associated with drug resistance.**

(A) Apoptosis was evaluated using the Annexin V/PI staining assay. (B) Cells were collected for cell cycle analysis by flow cytometry. (C) The expression of proteins associated with drug resistance was assessed by Western blot analysis. Data are presented as the mean ± SD from three independent experiments. Statistical analysis was conducted using one-way ANOVA. NS, not significant; ^*^*P* < 0.05, ^**^*P* < 0.01 and ^#^*P*<0.001.

**Supplementary Table 1. List of antibodies and suppliers used in the study**

| **Antibody** | **Isotype** | **Suppliers** | **Cat. no** | **Dilution factor** | **Application** |
| --- | --- | --- | --- | --- | --- |
| FOXA1 | Mouse | Santa Cruz Biotechnology | sc-101058 | 1:1000 | WB |
|  |  |  |  | 1:400 | IHC |
| BMI1 | Rabbit | abcam | ab71559 | 1:2000 | WB |
|  |  |  |  | 1:400 | IHC |
| H2AK119ub1 | Rabbit | Cell Signaling Technology | 8240T | 1:2000 | WB |
| GAPDH | Rabbit | Proteintech | 10494-1-AP | 1:5000 | WB |
| β-tubulin | Mouse | Affinity Biosciences | T0023 | 1:10000 | WB |
| MDR1 | Rabbit | Wanleibio | WL02395 | 1:2000 | WB |
|  |  |  |  | 1:400 | IHC |
| MRP1 | Rabbit | Wanleibio | WL01027 | 1:1000 | WB |
|  |  |  |  | 1:400 | IHC |
| FOXA1 | Rabbit | Cell Signaling Technology | 53528 | 1:50 | ChIP |
| Histone H3 | Rabbit | Cell Signaling Technology | 4620 | 1:50 | ChIP |
| IgG | Rabbit | Cell Signaling Technology | 2729 | 1:500 | ChIP |
