# Supplementary material for: Targeting the FOXA1/BMI1 axis to overcome chemoresistance and suppress tumor progression in nasopharyngeal carcinoma
Source: Cell Death Discov. 2025 Jul 7;11:311. doi: 10.1038/s41420-025-02595-6 (PMC12234731; doi:10.1038/s41420-025-02595-6)
Supplement: Supplementary file 2 — Original full length western blots [file 41420_2025_2595_MOESM2_ESM.doc]

**Original full length western blots**

**Fig.1E
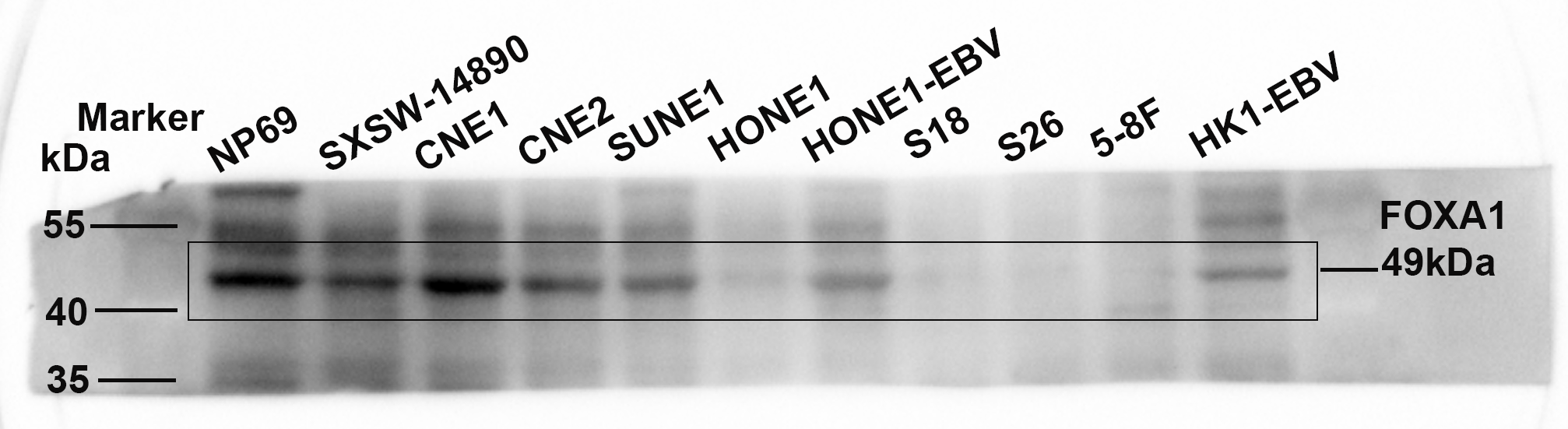
**

**FOXA1**

**
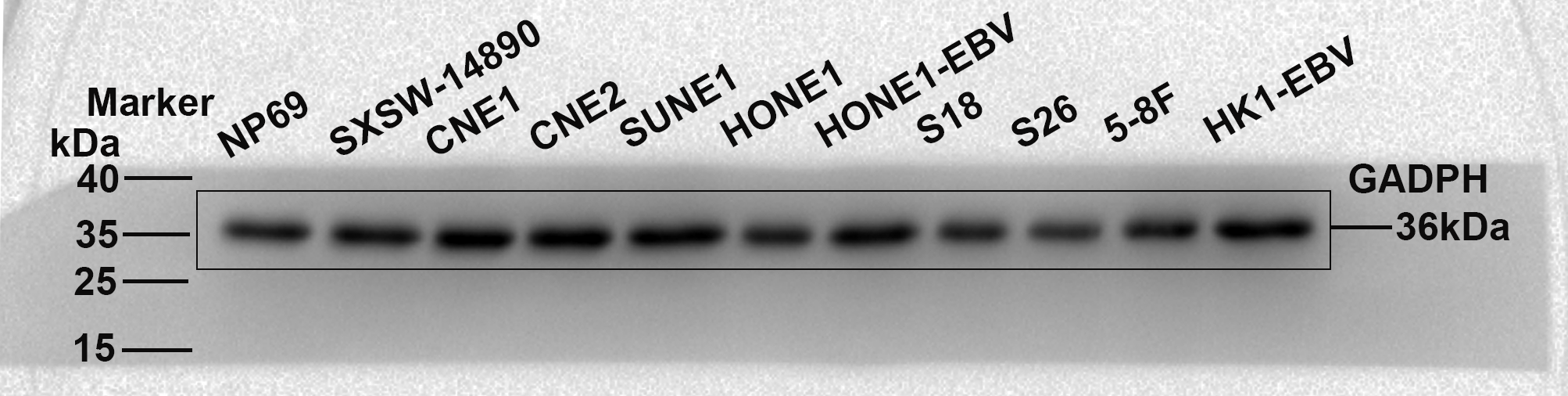
**

**GADPH**

**Fig.2A**


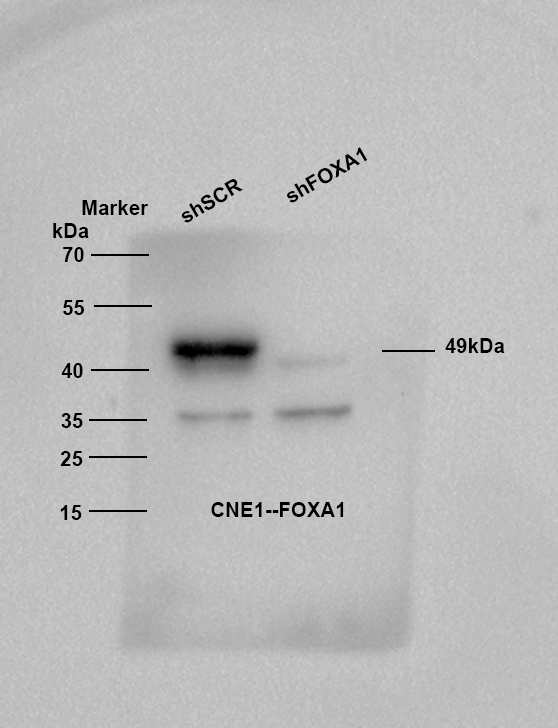

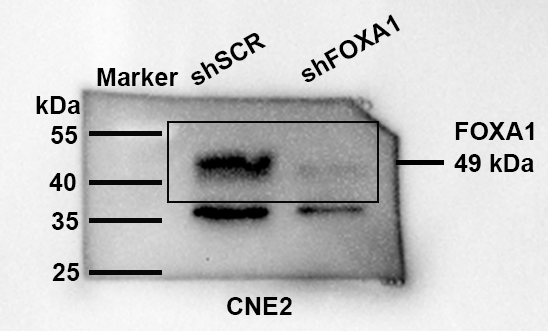


**CNE1-FOXA1 CNE2-FOXA1**


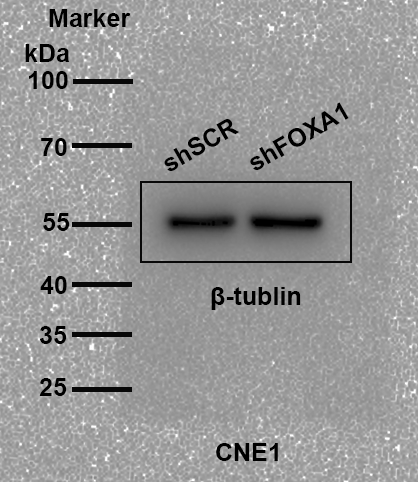

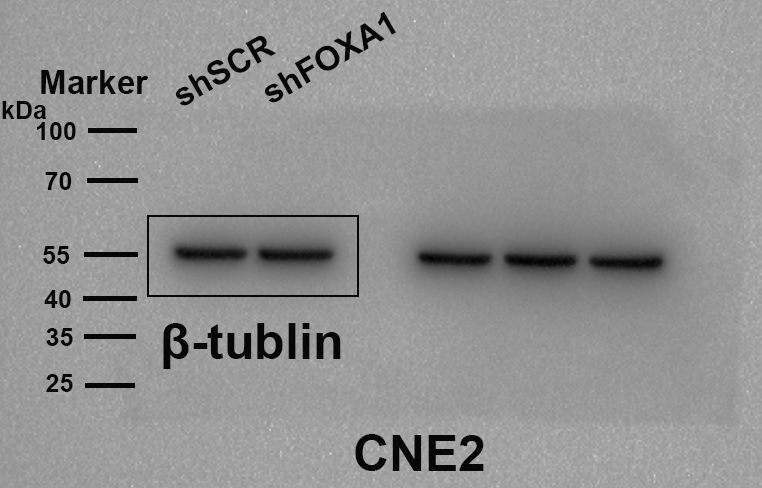


**CNE1-β-tublin CNE2-β-tublin**

**Fig.4B**


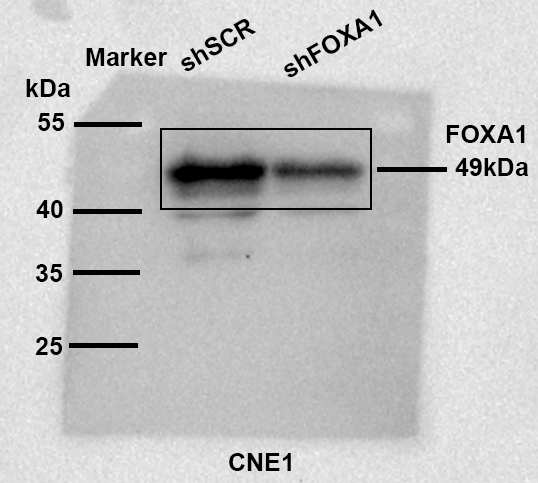

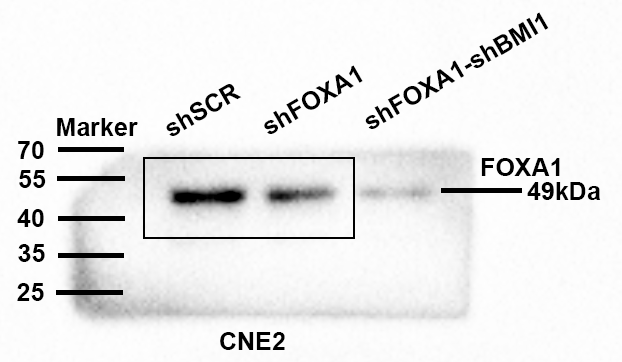


**CNE1-FOXA1 CNE2-FOXA1**


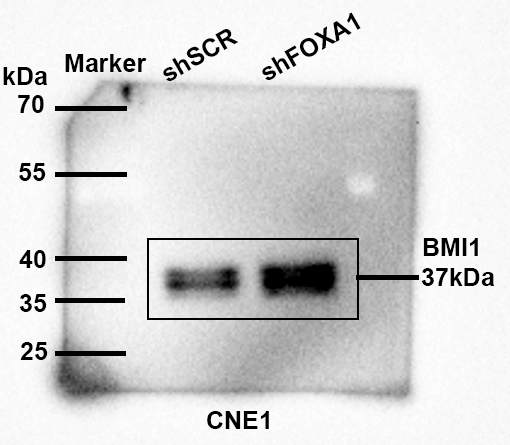

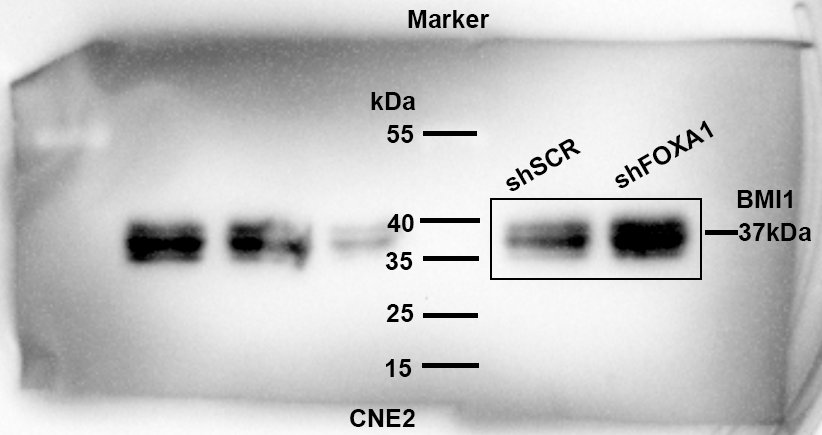


**CNE1-BMI1 CNE2-BMI1**


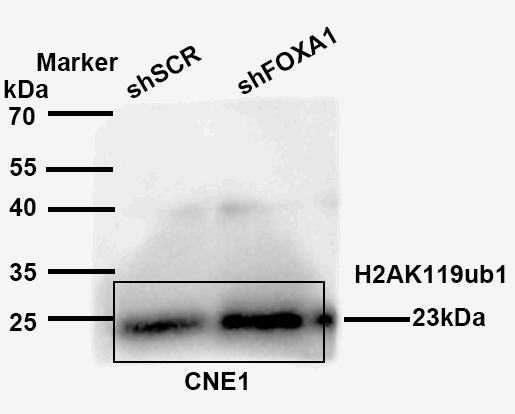

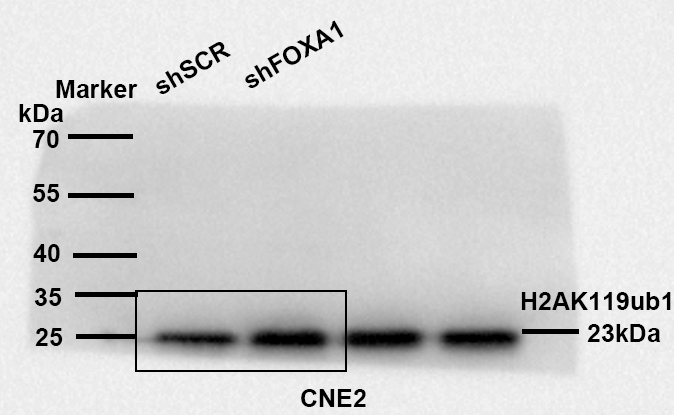


**CNE1-H2AK119ub1 CNE2-H2AK119ub1**


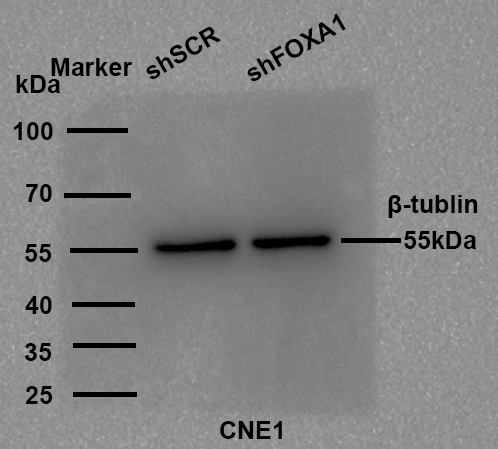

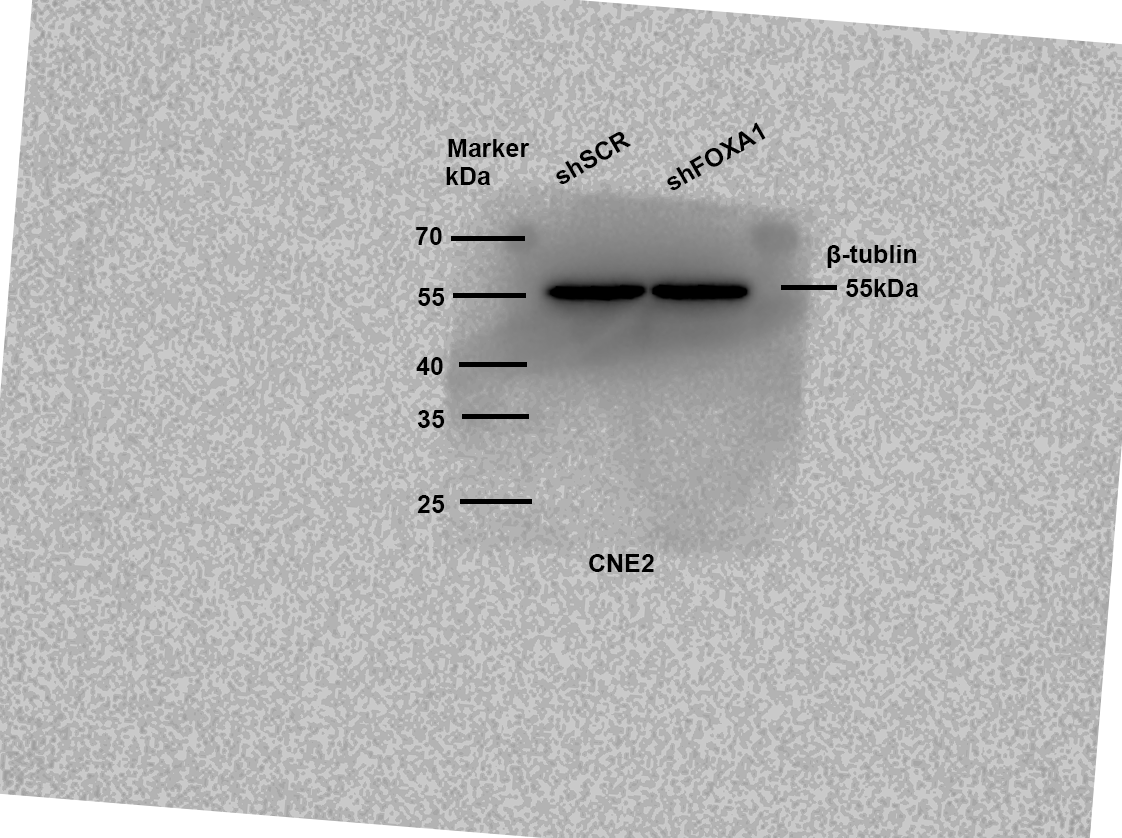


**CNE1-β-tublin CNE2-β-tublin**

**Fig.4E**


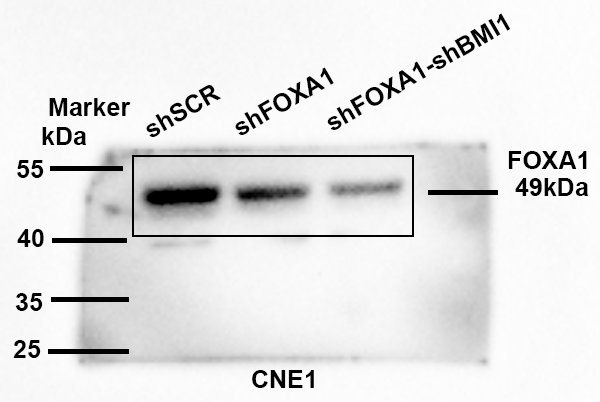

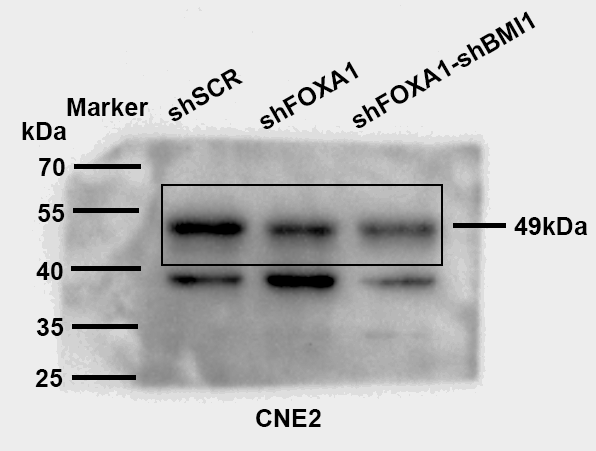


**CNE1-FOXA1 CNE2-FOXA1**


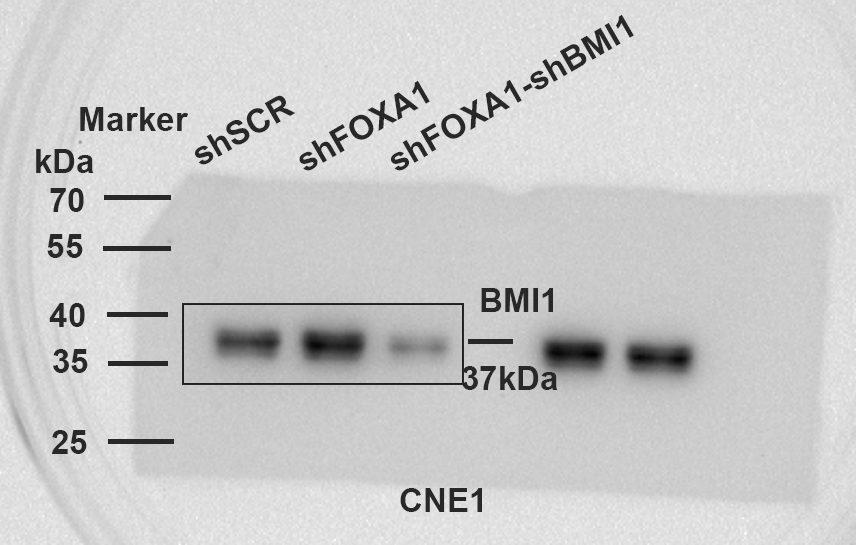

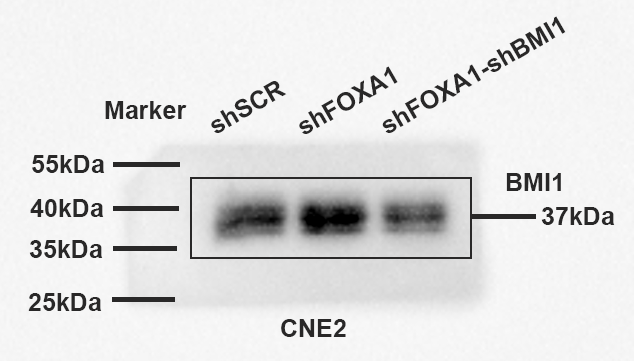


**CNE1-BMI1 CNE2-BMI1**


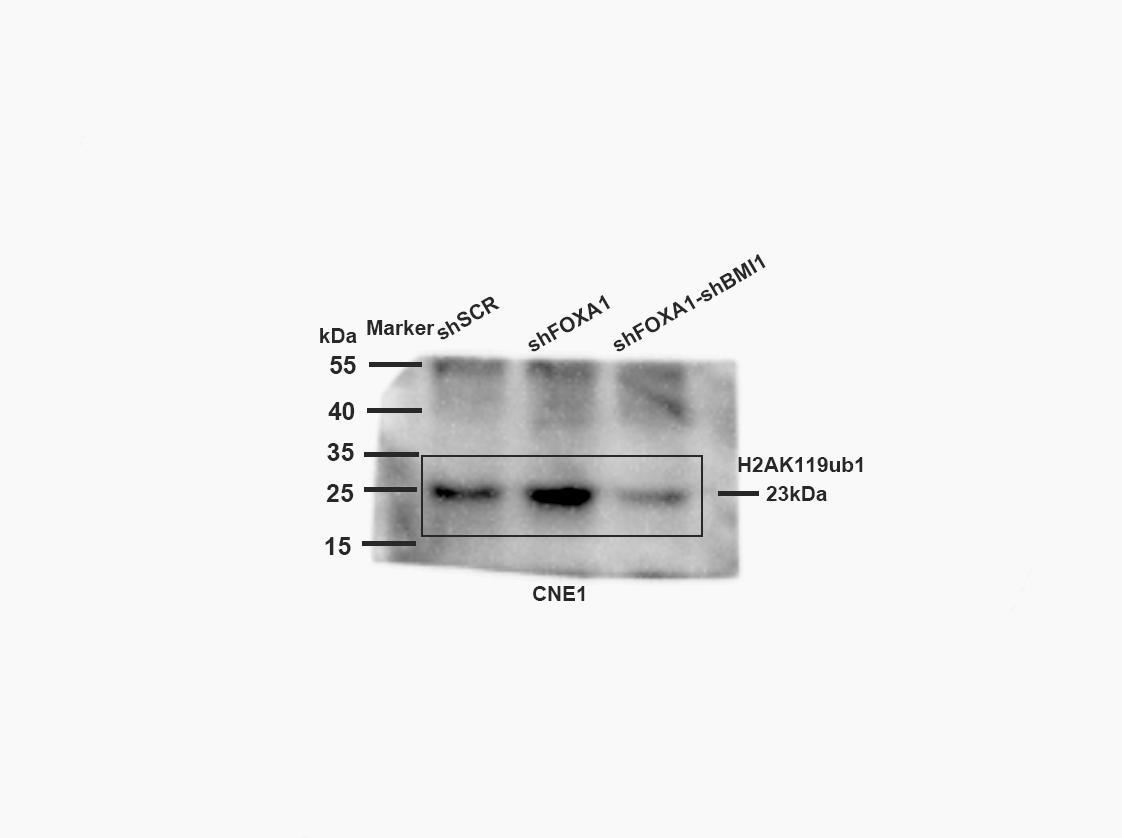

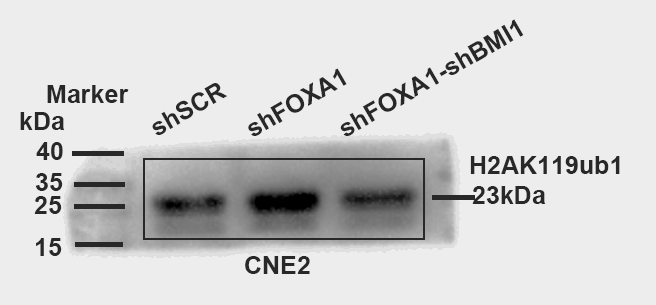


**CNE1-H2AK119ub1 CNE2-H2AK119ub1**


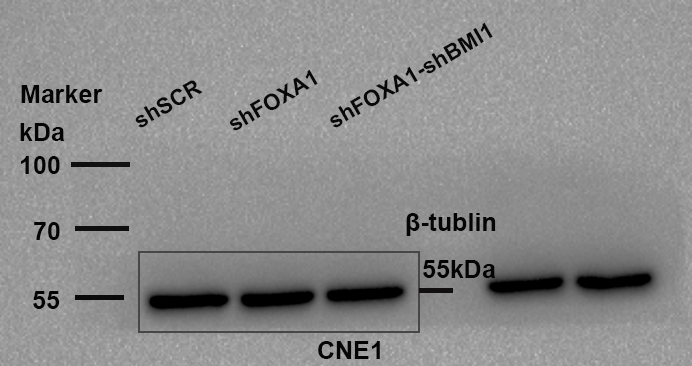

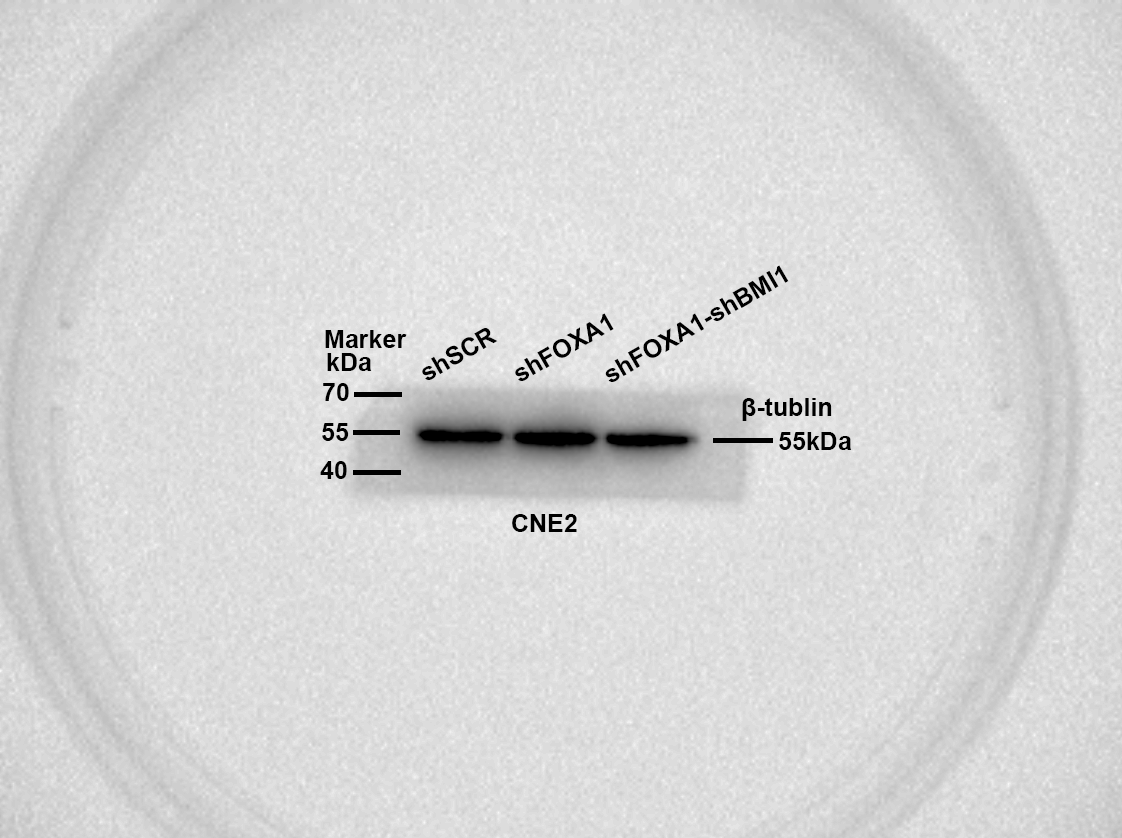


**CNE1-β-tublin CNE2-β-tublin**

**Fig.6E**


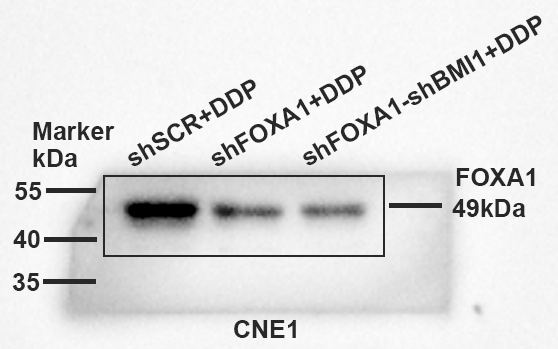

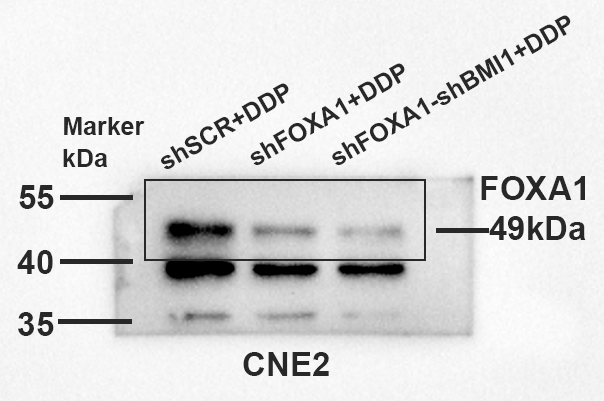


**CNE1-FOXA1 CNE2-FOXA1**


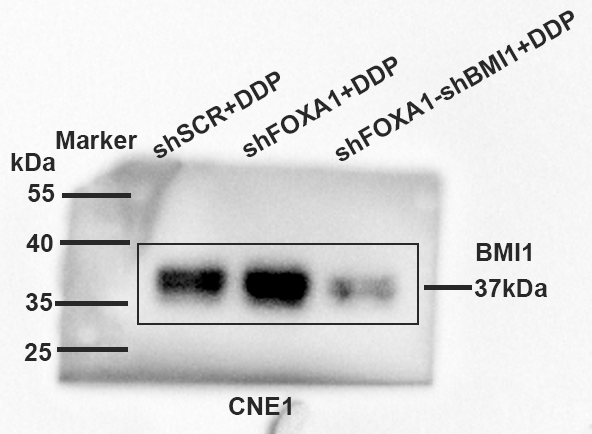

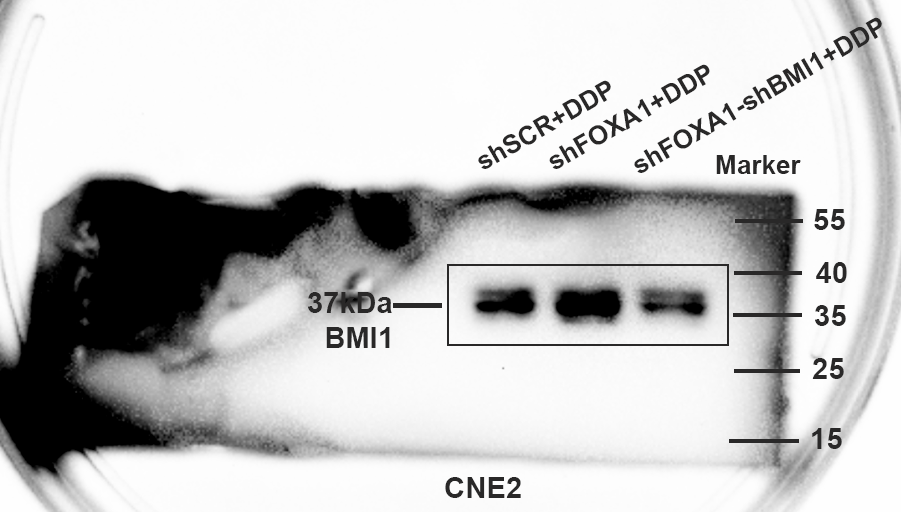


**CNE1-BMI1 CNE2-BMI1**


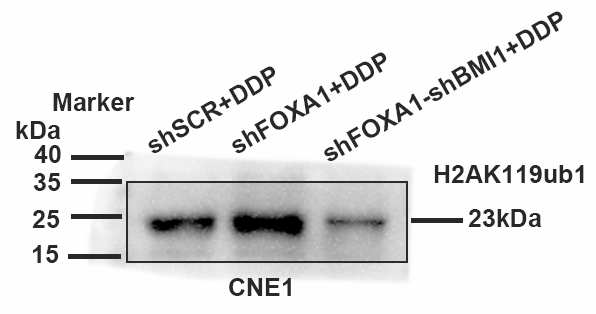

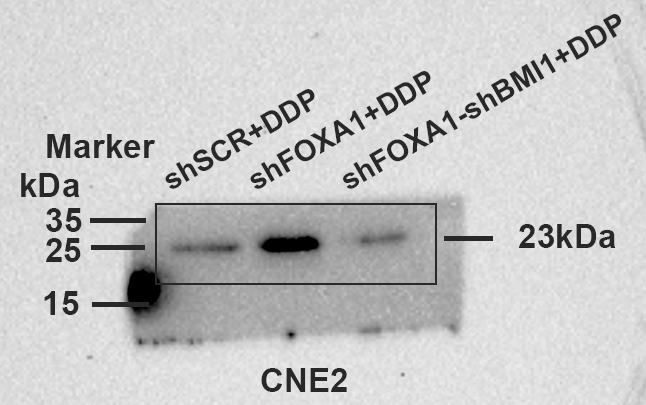


**CNE1-H2AK119ub1 CNE2-H2AK119ub1**


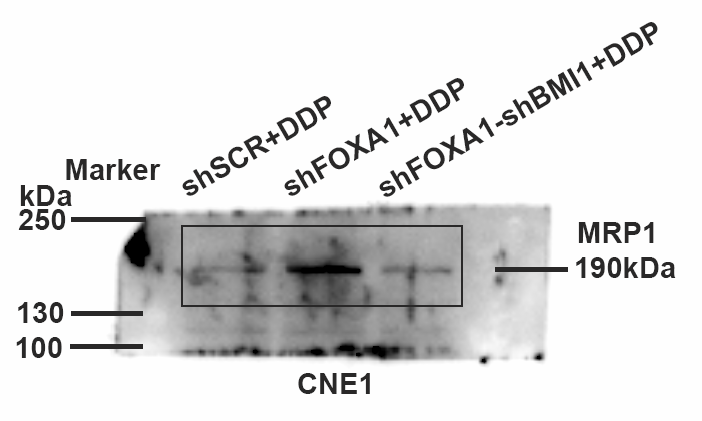

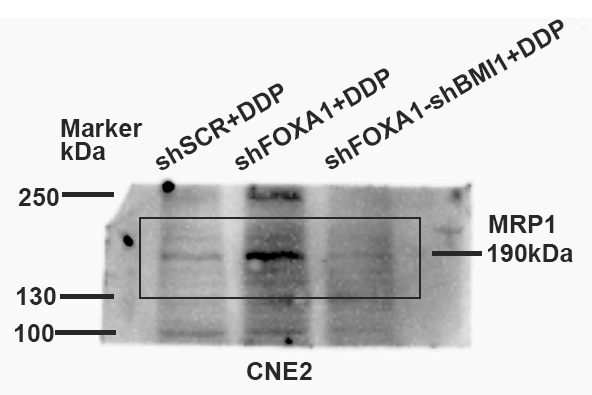


**CNE1-MRP1 CNE2-MRP1**


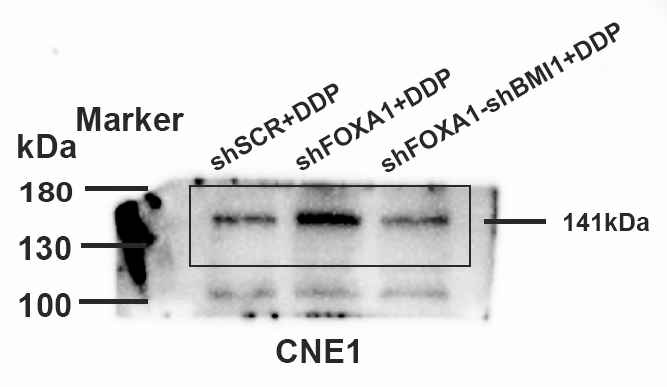

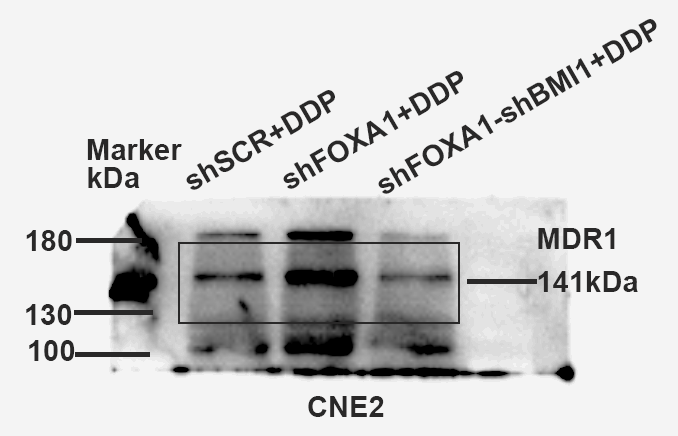


**CNE1-MDR1 CNE2-MDR1**


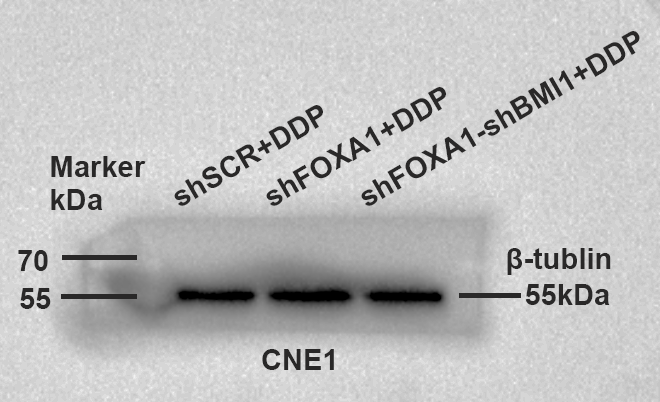

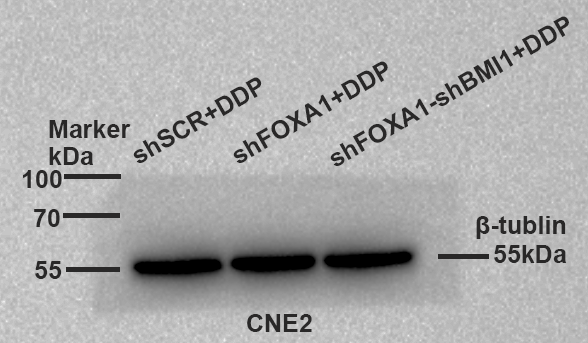


**CNE1-β-tublin CNE2-β-tublin**

**Supplementary Fig 2A**


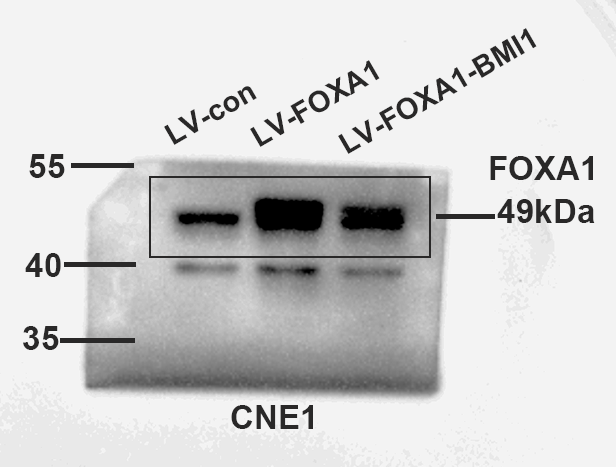

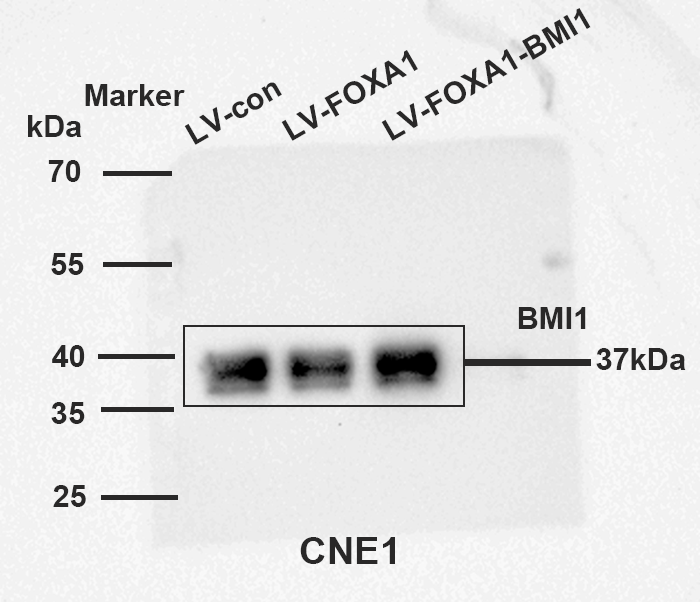


**FOXA1 BMI1**


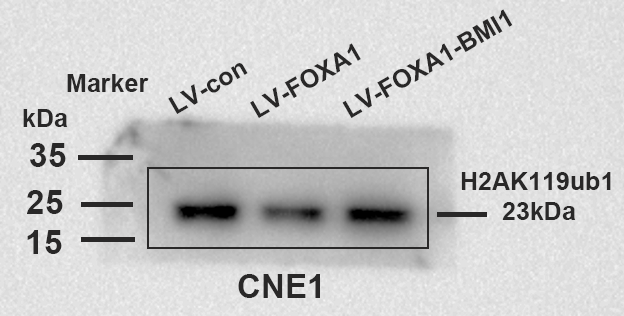

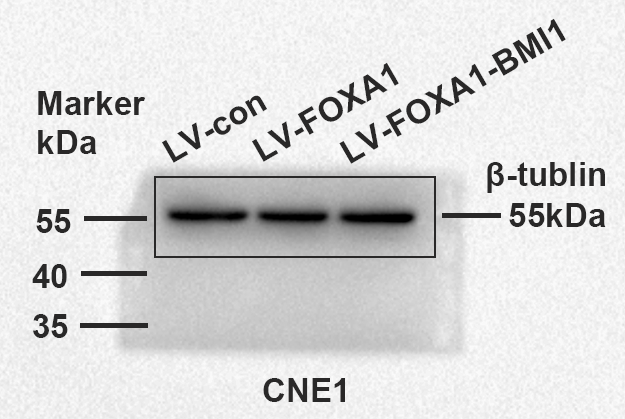


**H2AK119ub1 β-tublin**

**Supplementary Fig 4C**


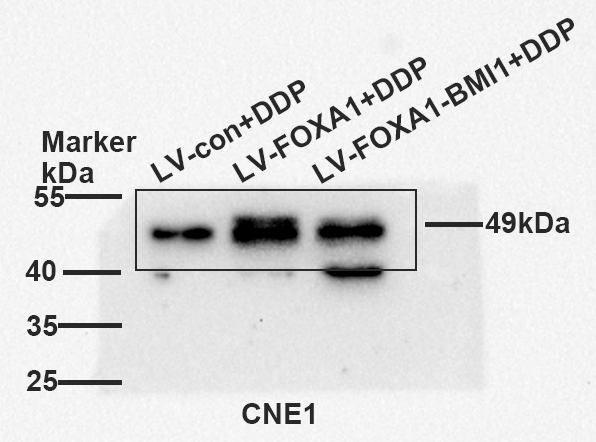

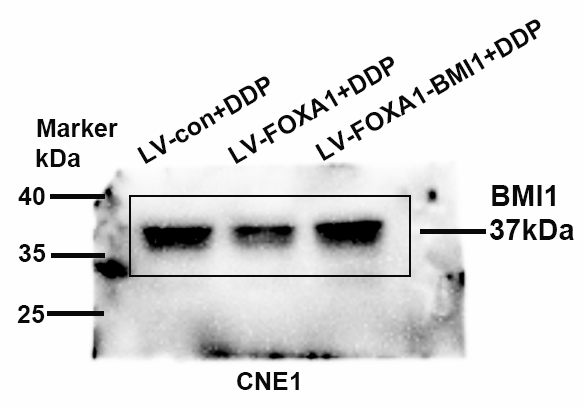


**FOXA1 BMI1**


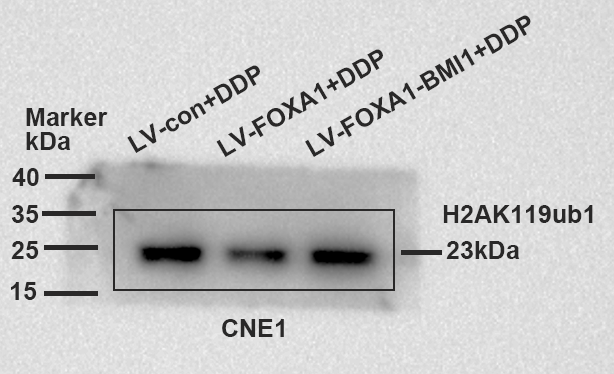

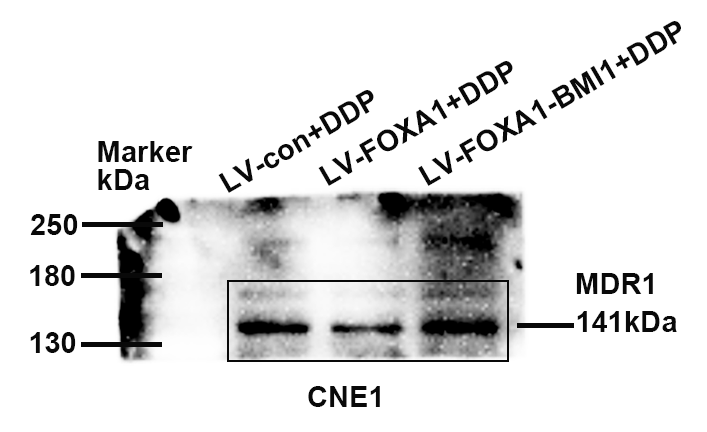


**H2AK119ub1 MDR1**


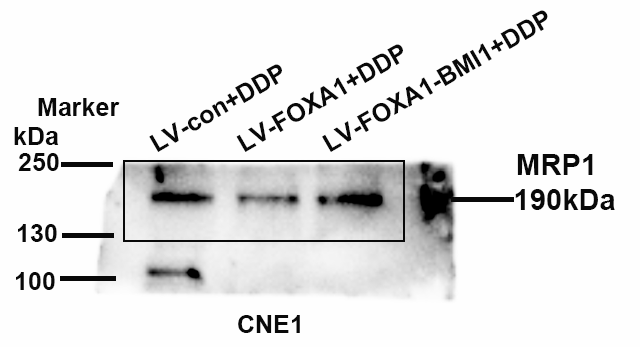

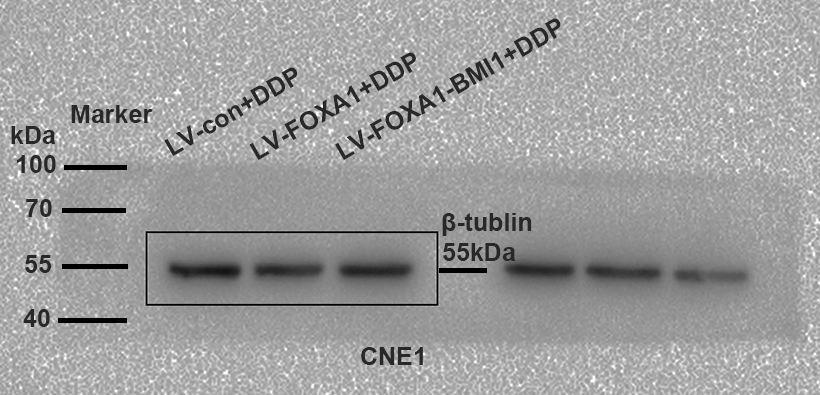


**MRP1 β-tublin**
